# Endoplasmic Reticulum Stress-Sensing Mechanism Is Activated in *Entamoeba histolytica* upon Treatment with Nitric Oxide

**DOI:** 10.1371/journal.pone.0031777

**Published:** 2012-02-24

**Authors:** Julien Santi-Rocca, Sherri Smith, Christian Weber, Erika Pineda, Chung-Chau Hon, Emma Saavedra, Alfonso Olivos-García, Sandrine Rousseau, Marie-Agnès Dillies, Jean-Yves Coppée, Nancy Guillén

**Affiliations:** 1 Institut Pasteur, Unité Biologie Cellulaire du Parasitisme, Paris, France; 2 INSERM U786, Paris, France; 3 Departamento de Bioquímica, Instituto Nacional de Cardiología, México DF, México; 4 Departamento de Medicina Experimental, Facultad de Medicina, Universidad Nacional Autónoma de México, México DF, México; 5 Institut Pasteur, Bioinformatics Group for Genomics Analysis, Genomes and Genetics Department, Paris, France; 6 Institut Pasteur, Genopole, Transcriptome and Epigenome Platform, Genomes and Genetics Department, Paris, France; Texas A&M University, United States of America

## Abstract

The Endoplasmic Reticulum stores calcium and is a site of protein synthesis and modification. Changes in ER homeostasis lead to stress responses with an activation of the unfolded protein response (UPR). The *Entamoeba histolytica* endomembrane system is simple compared to those of higher eukaryotes, as a canonical ER is not observed. During amoebiasis, an infection of the human intestine and liver by *E. histolytica*, nitric oxide (NO) triggers an apoptotic-like event preceded by an impairment of energy production and a loss of important parasite pathogenic features. We address the question of how this ancient eukaryote responds to stress induced by immune components (*i.e.* NO) and whether stress leads to ER changes and subsequently to an UPR. Gene expression analysis suggested that NO triggers stress responses marked by (i) dramatic up-regulation of *hsp* genes although a *bona fide* UPR is absent; (ii) induction of DNA repair and redox gene expression and iii) up-regulation of glycolysis-related gene expression. Enzymology approaches demonstrate that NO directly inhibits glycolysis and enhance cysteine synthase activity. Using live imaging and confocal microscopy we found that NO dramatically provokes extensive ER fragmentation. ER fission in *E. histolytica* appears as a protective response against stress, as it has been recently proposed for neuron self-defense during neurologic disorders. Chronic ER stress is also involved in metabolic diseases including diabetes, where NO production reduces ER calcium levels and activates cell death. Our data highlighted unique cellular responses of interest to understand the mechanisms of parasite death during amoebiasis.

## Introduction

Certain cell types of the innate immune system (*e.g*. macrophages, neutrophils and natural killer cells) are activated during infection by unicellular parasites, viruses or bacteria. These cells attack the pathogens by releasing a variety of effecter molecules, including nitric oxide (NO), which is an intracellular messenger known to be one of the most versatile players in the immune system [1], [2]. Activated macrophages synthesize high levels of NO that favor an interaction with reactive oxygen species (ROS) and production of peroxynitrite (ONOO^−^), a powerful radical that destroys cells and can stimulate apoptosis. Under physiological conditions, excess peroxynitrite (and other ROS) is prevented by antioxidant defense systems in the cells where proper protein folding and disulfide bond formation is dependent on the redox status within the endoplasmic reticulum (ER). Accumulating evidence has indicated that a crosstalk occurs between the generation of ROS and an ER stress response [3]. The ER is a dynamic organelle and changes in its size and components have been described, either as a result of changes in the secretory capacity of cells or as a result of adaptation to diverse stresses. Vigorous protein synthesis and increase in the levels of misfolded proteins which accumulate in the ER prompt a response known as the unfolded protein response (UPR) [4]. The purpose of the UPR is to restore normal ER function, relieve stress exerted on the ER, and prevent the cytotoxic impact of malformed proteins.

Amoebiasis is an infectious disease of humans caused by the protozoan parasite *Entamoeba histolytica.* Intestine and liver are the main sites of infection. Amoebic liver abscesses (ALAs) are the most frequent and severe extra-intestinal clinical manifestations of amoebiasis [5]. During the early stages of ALA formation, activated macrophages produce TNF, which promotes NO synthesis by macrophages and neutrophil PMNs [6], [7]. The comparison of gene expression profiles for virulent and avirulent parasites demonstrates that virulence traits are related to the expression of stress responses in *E. histolytica* and should be linked to UPR activation upon exposure of parasites to inflammation [8]. An interesting aspect of *E. histolytica* cellular biology is that this single-celled organism lacks visible classical organelles such as mitochondria and rough ER although it has Golgi/ER associated functions and contains remnant mitosomes where the major function in *E. histolytica* is sulfate activation [9]. Typically, the ER is organized into interconnected branching tubules and flattened sacs. Resident luminal ER proteins containing the KDEL sequence motif are retained in the lumen of the ER. A recently engineered plasmid construct, introduced in *E. histolytica*, generates trophozoites expressing a hybrid protein containing a FLAG tag, the green fluorescent protein (GFP) and the KDEL retrieval motif [10]. The GFP-KDEL-FLAG was shown to localize in a continuous intracellular compartment and to colocalize with the BiP (Hsp70) ER resident chaperone.

The viability of *E. histolytica* is compromised by the presence of NO, which is demonstrated by massive parasite deaths *via* an apoptosis-like mechanism after 24 h of co-incubation with the NO donor sodium nitroprusside (SNP) [11]. Functional assays of NO-treated parasites have shown a loss of certain amoebic virulence traits (e.g. hemolytic activity and complement resistance), whereas others are maintained (erythrophagocytosis and proteolytic activity) [11]. To gain better insights into both the ER structural dynamics and the effect of NO on *E. histolytica*, we tested whether stress induced by immune effectors (i.e. NO) lead to ER stress and the subsequent UPR. Imaging of parasites treated with NO showed that the atypical ER in *E. histolytica* responds to NO and undergoes dramatic morphological modifications with the generation of vesicle-like structures. We examined amoebic gene expression during incubation of growing parasites with NO. The data revealed gene expression patterns associated with membrane traffic, extreme stress responses, cysteine metabolism and anaerobic energy production. Further biochemical assays determined that the activity of four enzymes involved in ethanol production, including pyruvate:ferredoxin oxidoreductase (PFOR), bifunctional aldehyde-alcohol dehydrogenase (NAD+-ADH), malic enzyme and NADP+-alcohol dehydrogenase (NADP+-ADH), were strongly inhibited inhibited whereas cysteine synthase activity was increased in amoeba treated with SNP. Our study has implications for understanding the biology of *E. histolytica* during parasitic infection since ROS accumulation induces both oxidative and ER stress, which lead to glycolysis dysfunction and parasite death.

## Results

### Incidence of nitric oxide treatment in endoplasmic reticulum organization

**Figure 1:**
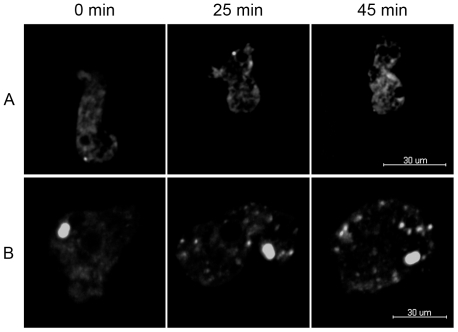
Live time-lapse imaging of *E. histolytica* endoplasmic reticulum during NO treatment. GFP-KDEL-FLAG transfected amoebas were embedded in a type I collagen matrix treated or not with SNP. One set of images was taken every minutes for an hour. Each stack was acquired using a 0.5 µm Z step. Time points are shown for 0, 25 and 45 min after treatment for both untreated (A) and treated (B) samples. During treatment, the normal organization of the ER begins to change around 25 min after treatment and breaks into vesicles that are found throughout the cytosol by 45 min. Scale bar equals 20 µm.

Although NO is clearly an important stress factor for *E. histolytica*, nothing is known about how this stress affects the endoplasmic reticulum (ER), an organelle that is typically known to house the UPR elements during extreme cellular stress. Therefore, we assessed the effects of NO stress on the ER of *E. histolytica*. The morphology of the ER in trophozoites incubated in the presence of SNP, a rapid thiol-dependent NO donor, was analyzed by taking advantage of a GFP-KDEL-FLAG construct, which has been shown to localize to the ER. First, we examined the effects of NO on *E. histolytica* in real time by imaging the ER morphology changes. To maintain parasite adherence, we embedded the trophozoites in a type I bovine collagen matrix, which provides a stable 3D environmental platform to image highly motile *E. histolytica*. For the NO treated samples, SNP was added on the top of each matrix before image acquisition. We showed that in the untreated matrices, GFP-KDEL-FLAG staining revealed a perinuclear intracellular compartment that appeared to be continuous through the cytoplasm. The labeling remained constant over the course of the experiment (Figure 1 and Video S1). In contrast with the untreated samples, we observed dramatic changes of ER morphology in the NO treated samples. Initially, the localization of GFP-KDEL-FLAG was similar to the untreated and treated samples. Around 25 min after treatment, we observe that the ER was broken into vesicle-like structures throughout the cytoplasm, which correlates with the disappearance of the perinuclear ER and the loss of a continuous ER structure (Figure 1 and Video S2).

**Figure 2:**
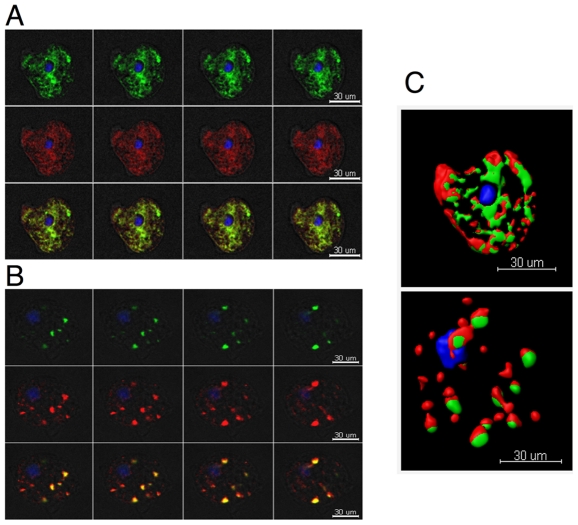
Nitric oxide treatment effects on *E. histolytica* endoplasmic reticulum with respect to GFP-KDEL-FLAG and calreticulin. Amoebas were treated with SNP and then fixed and labeled with both anti-FLAG and anti-CRT. Four confocal planes are shown for both untreated (A) and treated (B) samples. GFP-KDEL-FLAG (green), CRT (red), and DAPI (blue). The images show that NO treatment dramatically changes the ER and affects multiple ER markers. The two markers are localized to the same compartment in both treated and untreated samples. Images were analyzed using the iMARIS software to generate 3D models (C). The top panel (C) shows the model of the untreated amoeba from A and the bottom panel depicts the model of the treated amoeba from B. Scale bars equal 30 µm.

**Figure 3:**
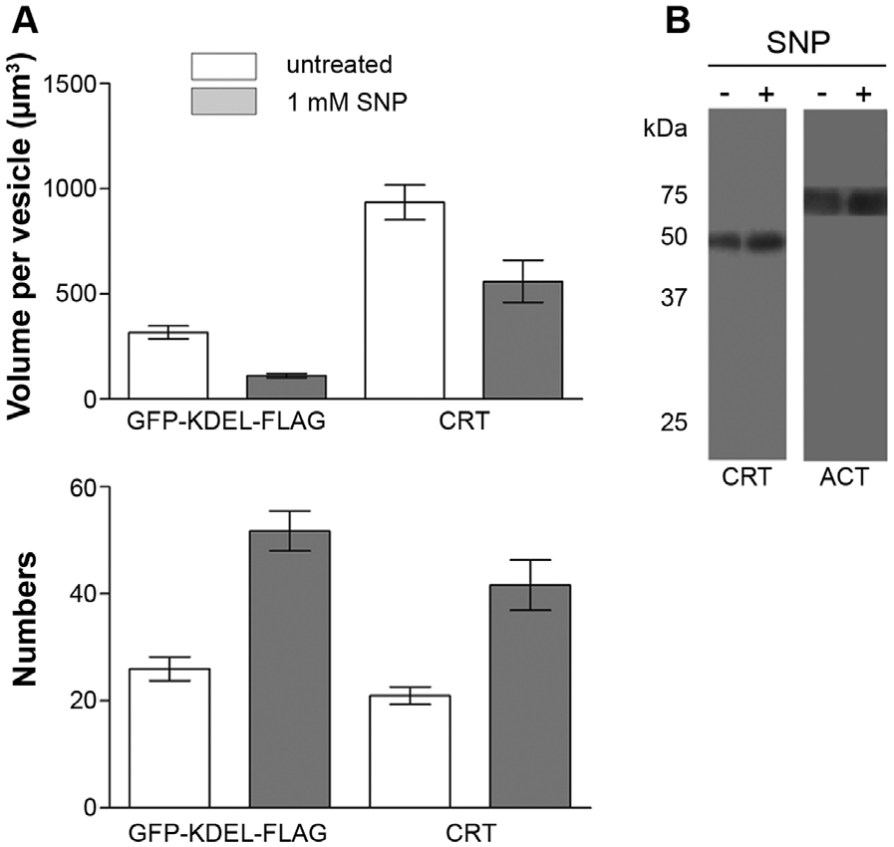
Quantification of ER changes after nitric oxide treatment. The nitric oxide induced changes were quantified using the iMARIS software. An average of 30 amoebas were analyzed per experiment. The total number of labeled compartments and the volume per labeled compartment were determined. The data show that both ER markers, KDEL and CRT, exhibit a similar change with a decrease in the volume of labeled vesicles and an increase in the number of vesicles present (A) . An unpaired t-test was performed and a statistically significant difference was found between treated and untreated samples for both ER markers (** p<0.05 and *** p<0.01). The calreticulin protein level was analyzed, 40,000 amoebas were loaded in each lane for each condition. An anti-actinin-2 antibody was used to control for equal loading of samples. No changes in the calreticulin protein levels were observed. Although there are changes in ER organization, the protein levels remain constant (B). Three independent experiments were conducted.

Following our initial time course studies, we next investigated whether the observed localization changes of GFP-KDEL-FLAG were similar to that of calreticulin (CRT), which is another known ER marker. As it has been suggested that there are separate and distinct ER compartments in the amoeba, it is therefore important to determine how GFP-KDEL-FLAG and the associated stress changes were correlated to that of CRT, a protein that buffers Calcium concentrations in the lumen of the ER. An average of 30 amoebas per experiment per treatment (+/− SNP) was examined by immunofluorescence and confocal microscopy. The analysis showed that both GFP-KDEL-FLAG and CRT localized in the same ER compartment, as is evidenced by the co-staining observed (Figure 2) in both untreated and treated samples. We observed that under NO stress there were equivalent changes in CRT staining as that observed with the GFP-KDEL-FLAG samples. Instead of being a continuous organelle around the nucleus and throughout the cytoplasm, fragmentation of the ER was also confirmed by the dispersion of CRT labeling. Using the Imaris software, we were able to determine the numbers of labeled compartments along with the volume of these compartments. The volume per staining vesicle of the GFP-KDEL-FLAG was shown to decrease from around 300 µm^3^ to 100 µm^3^, while the number of vesicles increased from 25 to 50 vesicles (Figure 3A). Calreticulin showed a decrease in volume per vesicle from around 930 µm^3^ to 560 µm^3^, with an increased number of vesicles from 20 to 42 (Figure 3A). Under NO stress, both GFP-KDEL-FLAG and CRT labeled compartments were fragmented into small vesicles, and even under stress, they remained localized together. In addition, we also investigated the changes of CRT protein level during the stress responses using western blot analysis. Anti-actinin-2 antibody was used as a control. No significant difference in the abundance of the CRT protein was observed under stress (Figure 3B).

### Gene expression modulation in response to NO treatment

To identify the differentially regulated genes induced by NO treatment, we chose to perform microarray analysis on the transcriptomes of trophozoites after 1 h treatment with NO (at the same time point as for the imaging experiments). Previous experiments have examined *in vitro* non-adherent parasite viability following treatment with the NO donor SNP [11]. We checked that adherent cultured parasites incubated with 1 mM SNP underwent cytotoxic shock. After a 1 h incubation with 1 mM SNP and 24 h recovery post-incubation, we counted 47.67% (CI_α = 0.05_ = [41.28%; 54.08%]) of live, adherent parasites, compared with untreated amoeba. Early responses to NO were identified by comparing the transcriptomes of adherent, virulent trophozoites incubated in the presence or absence of 1 mM SNP for 1 h without the post-incubation recovery.

To accommodate the latest version of *E. histolytica* genome annotation, we designed new microarrays (EH-IP2008) that were validated with our previous *bona fide* chip (EH-IP2007), which had been validated by qRT-PCR under different conditions [8], [12]–[14]. To this goal, we identified changes in gene expression in response to NO, and compared the results obtained with the two versions of the microarray. By comparing the Log_2_ ratio of genes obtained with both chip versions, we showed a strong positive linear relationship between the two datasets (R^2^ = 0.85; Figure S1). For further analysis, we focused on genes with a fold-change greater than 2 and a significant *p* value (≤0.05), we didn't consider hypothetical proteins in this analysis. For complete experimental details see Material and Methods section. We found that 365 genes were upregulated in the presence of NO, whereas 103 genes were downregulated (Table S1 and Table S2). Functional overviews of the up- and down-regulated genes were summarized using GO Slim Terms based on the GO annotations in AmoebaDB version 1.3 (Table S3). These overviews shown changes in cellular process involved in macromolecules binding, oxidoreduction and hydrolysis reactions among the most highly modulated genes. To facilitate the biological interpretations of microarray results, we developed a desktop application for rapid retrieval of the functional annotations concerning the differentially expressed genes. This application enables us to manually curate the potential biological functions and the orthologies of the differentially expressed genes in an efficient manner. It is noteworthy that the proteins discussed in the following paragraphs were all manually curated using the novel desktop application.

### Treatment with nitric oxide modifies expression of *E.histolytica* genes encoding factors responsible for changes in the dynamics of endomembrane traffic

In human cells, ER stress caused by NO treatment modifies the dynamics of endomembrane transport [15]. Due to the phenotype we observed by microscopy, we sought a change in the expression of genes responsible for endomembrane trafficking in the parasite, which could participate in the alteration of ER morphology. Interestingly, the gene encoding ARF GTPase-activating protein (EHI_012490) was upregulated. In human cells, inhibition of ARF GTPase activity leads to fission of the Golgi apparatus and retrograde transport to this organelle [16]. Moreover, we identified an overexpressed gene encoding a homolog of the vacuolar protein sorting subunit 26, Vps26 (EHI_078250), a protein of the retromer complex that in *E. histolytica* plays a role in vesicular transport [17]. The modification in expression of genes encoding proteins involved in anterograde and retrograde vesicle transport suggests that NO disrupts the equilibrium in endomembrane trafficking.

### An unfolded protein response during stress is absent in *E. histolytica*

Given that NO is known to induce stresses leading to cell death in *E. histolytica*, a more detailed examination and description was performed for the functional classes that could give information about the modifications in trophozoite physiology through genes involved in UPR, DNA repair, redox activities and carbohydrate metabolism.

UPR is a response to ER stress that aims to recover the native conformation of the degrading unfolded proteins that are accumulated in this organelle. Cellular factors involved in UPR are the Inositol-Requiring Enzyme 1 (IRE1), a transmembrane Protein kinase R-like Endoplasmic Reticulum Kinase, (PERK), which upon phosphorylation inactivates protein translation initiation factor 2a (eIF2α) and activating transcription factor 6 (ATF6). These three proteins interact with the chaperone BiP (i.e. Hsp70) whose over-expression attenuates the UPR. We found that *E. histolytica* genome does not encode obvious orthologues of PERK or ATF6, however the expression of the gene encoding eIF2α (EHI_005100) was upregulated.

From the 365 upregulated genes, 83 of them fall into the category of the so-called heat shock proteins (Hsps), which form a broad group of molecular chaperones, whose production is upregulated upon exposure to high temperature and various other stresses. Strikingly, *hsp* genes were highly upregulated when trophozoites were incubated with SNP (Table S4); these include the genes encoding the high temperature tolerance Hsp101 (named Hsp104 in yeast) and the Caseinolytic peptidase B protein (ClpB), the chaperones Hsp90, Hsp70, Hsp20, the heat shock-cognate 70 kDa-interacting protein (Hip, which has been identified as an anti-apoptotic protein [18]), and the 70 kDa peptidyl-prolyl isomerase (PPI) which, together with Hsp70 and Hsp90, is a component of the N-glycan-independent protein folding quality control system within the lumen of the ER (9). The gene encoding the chaperonin containing TCP-1 zeta subunit was also upregulated and is part of a protein folding complex. It is noted that no genes of this functional group were suppressed upon NO stress, thus reinforcing the expected significance of the HSPs during this stress. Notice that we did not find significant modification of transcription for genes encoding Hsp transcription factors (EHI_008660, EHI_008230, EHI_087630 and EHI_200020) or CRT.

### Expression of genes encoding anti-oxidative enzymes involved in redox reactions is modulated by nitric oxide treatment

*Entamoeba histolytica* lacks mitochondria and the usual aerobic respiratory pathways. This parasite is generally considered as anaerobic/microaerophilic and has been shown to consume oxygen (not associated with ATP synthesis) and tolerate low levels of oxygen pressure but lacks most of the components of antioxidant defense mechanisms, such as catalase, peroxidase, glutathione, and the glutathione-recycling enzymes whereas it contains activities for superoxide dismutase and thioredoxin reductase (for a review [19] and references herein). The analysis revealed a modulation of the expression of genes encoding anti-oxidant enzymes. One gene encoding peroxiredoxin, which is responsible for detoxification of hydrogen peroxide (Table 1), and three genes coding for iron-sulfur flavoproteins (types B and D) were upregulated. Thioredoxin and oxidoreductases such as NAD(P)^+^-binding protein and disulfide isomerase were down-regulated. These observations imply that important changes in anti-oxidative pathways in trophozoites may have occurred upon NO stress.

**Table 1:** Genes modulated by no linked to redox activities.

GENE ID	Description	FC	BY	rawp
**UP**				
EHI_138480	iron-sulfur flavoprotein, FprB2	7.3	2.4E-04	4.8E-07
EHI_025710	iron-sulfur flavoprotein, FprD3	4.2	2.6E-04	6.4E-07
EHI_032670	iron-sulfur flavoprotein, FprD1	2.9	2.4E-04	4.8E-07
EHI_159640	iron hydrogenase	2.6	2.3E-04	4.1E-07
EHI_018740	peroxiredoxin family	2.2	6.4E-04	8.0E-06
EHI_136380	cysteine desulfurase NifS	4.3	2.6E-04	6.5E-07
EHI_160930	cysteine synthase CysK	5,2	1.7E-04	8.3E-08
EHI_171750	cysteine synthase 2	4.0	6.1E-04	7.2E-06
EHI_060340	cysteine synthase A CysK	3.4	5.6E-04	5.9E-06
EHI_049620	Fe-S cluster assembly protein NifU	2.0	8.1E-03	3.6E-04
EHI_099700	NAD(FAD)-dependent dehydrogenase	3.9	3.9E-04	2.4E-06
**DOWN**				
EHI_062790	thioredoxin	0.3	6.0E-04	7.1E-06
EHI_133970	disulfide isomerase	0.5	3.6E-04	2.0E-06
EHI_125740	oxidoreductase	0.5	6.3E-04	7.7E-06

L-cysteine (Cys), which replaces glutathione as a major thiol in *E. histolytica*, is synthesized via a pathway consisting of two steps catalyzed by serine acetyltransferase and cysteine synthase. In addition, Cys provides an inorganic sulfur atom for the biosynthesis of Fe-S clusters, which are structural components of proteins involved in oxidative phosphorylation, electron transfer, and regulation of gene expression and of a variety of enzyme activities. In view of their ability to mediate redox reactions, Fe-S cluster proteins perform very diverse cellular functions. In bacteria, the nitrogen fixation (Nif) system is involved in the biosynthesis of Fe-S clusters. The only eukaryotic organism to use this system as its sole means of synthesizing Fe-S clusters is *E. histolytica* [20]. A number of hypotheses concerning the role of NifU have been made; in particular, it may act as a scaffold for Fe-S cluster formation, prior to the latter's transfer to target apoproteins [21]. Here, the *NifU* gene and the gene encoding the NifS desulphurase protein (which provides NifU with sulphide, S^2−^) were over-expressed in the presence of SNP. The L-cysteine required for this reaction can be supplied by cysteine synthase 2, whose gene was also upregulated. In parasites treated with SNP, we also detected the up-regulation of several open reading frames (ORFs) encoding Fe-S cluster-containing proteins potentially involved in oxidative stress protection and metabolism. For instance numerous genes encoding proteins involved in DNA repair were upregulated, rendering a probable gain of function for this mechanism (Table S4). The importance of DNA repair for cell survival in response to genotoxic stress together with the data presented above strongly suggests that this mechanism is part of the response of *E. histolytica* to NO.

### Expression of genes from the glycolytic pathway is upregulated by treatment with nitric oxide

*Entamoeba histolytica* lacks a functional tricarboxylic acid cycle and oxidative phosphorylation. Glycolysis and amino acid catabolism are known to be the major pathways for energy production in *E. histolytica* and pyruvate generation is one of the common steps [22]. After incubation with SNP, genes encoding five enzymes in the glycolytic pathway were upregulated and include glucose-6-phosphate isomerase (G6PI), glyceraldehyde-3-phosphate dehydrogenase (GAPDH), 2,3-biphosphoglycerate-independent 3-phosphoglycerate mutase (PGAM), enolase (ENO) and pyruvate phosphate dikinase (PPDK), which lead to the generation of pyruvate (Table 2). *E. histolytica* converts pyruvate to acetyl coenzyme A (acetyl-CoA) and carbon dioxide (CO_2_) in a reaction catalyzed by PFOR (whose encoding gene was upregulated), which transfers electrons to ferredoxin. Acetyl-CoA can be converted to acetate with production of ATP by acetyl-CoA synthetase (whose gene was upregulated) or to acetaldehyde and then ethanol by alcohol dehydrogenases (ADH), with oxidation of NAD(P)H. The genes encoding the NADH-dependent bifunctional aldehyde-alcohol dehydrogenase (ADH2), and NADPH-dependent alcohol dehydrogenase (ADH3) and other putative alcohol dehydrogenases (aldose reductase, NADPH-dependent oxidoreductases) were also over-expressed after exposure to SNP.

**Table 2:** Genes upregulated encoding metabolism enzymes.

GENE ID	Description	FC	BY	Rawp
EHI_157010	aldose reductase, NADPH-dependent oxidoreductase	5.9	2.9E-04	1.0E-06
EHI_107560	aldose reductase, NADPH-dependent oxidoreductase	5.9	3.8E-04	2.2E-06
EHI_039190	aldose reductase, NADPH-dependent oxidoreductase	5.4	2.0E-03	5.5E-05
EHI_024240	aldehyde-alcohol dehydrogenase ADH2	4.0	2.5E-04	5.9E-07
EHI_161010	glyceraldehyde-3-phosphate dehydrogenase GAPDH	3.5	3.5E-04	1.9E-06
EHI_160940	aldehyde-alcohol dehydrogenase ADH2	3.5	2.4E-04	4.5E-07
EHI_150490	aldehyde-alcohol dehydrogenase ADH2	3.2	3.7E-04	2.1E-06
EHI_050940	2,3-bisphosphoglycerate mutase iPGAM	3.1	2.7E-04	8.2E-07
EHI_009530	pyruvate phosphate dikinase PPDK	3.0	5.6E-05	4.7E-09
EHI_198760	alcohol dehydrogenase ADH3	2.9	3.0E-04	1.2E-06
EHI_051060	pyruvate:ferredoxin oxidoreductase PFOR	2.8	5.5E-05	3.8E-09
EHI_045080	pyruvate phosphate dikinase PPDK	2.6	5.8E-04	6.2E-06
EHI_088020	alcohol dehydrogenase ADH3	2.5	3.8E-04	2.3E-06
EHI_045090	pyruvate phosphate dikinase PPDK	2.3	3.6E-04	1.9E-06
EHI_130700	enolase ENO	2.3	3.5E-04	1.8E-06
EHI_030180	alcohol dehydrogenase ADH3	2.2	5.3E-04	5.2E-06
EHI_125700	dTDP-D-glucose 4,6-dehydratase	2.2	2.7E-04	8.5E-07
EHI_178960	acetyl-CoA synthetase	2.2	5.3E-04	5.2E-06
EHI_044970	malic enzyme	2.1	5.2E-04	5.0E-06
EHI_047730	glucose-6-phosphate isomerase PGI	2.1	3.1E-04	1.3E-06
EHI_072240	glucose-6-phosphate isomerase PGI	2.0	2.7E-04	7.1E-07

### Energy production-linked enzymatic activities inSNP-treated *E. histolytica*

**Figure 4:**
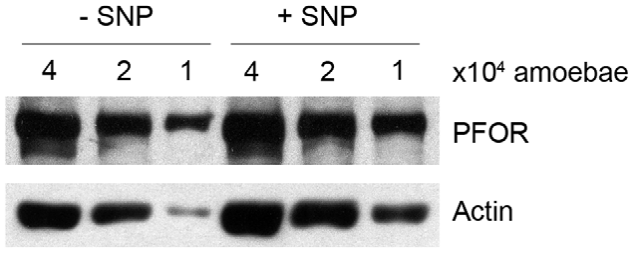
Immunodetection of PFOR levels upon SNP treatment. PFOR of 120 kDa was revealed upon SDS-PAGE and immunobloting of crude extracts from SNP-treated and control trophozoites (4, 2 and 1×10^4^ cells). The loaded amount of proteins for each condition is evidenced by actin (43 kDa) immunodetection on the same western blot. Quantification of signal emission did not reveal differences between the tested conditions in 3 independent experiments.

Previous data suggest that in some cases glucose can amplify ER stress [23]. Moreover, amoeba subjected to a supraphysiological level of oxygen, which induces increased ROS levels, showed important changes in glycolytic/fermenting enzyme activities and metabolic fluxes [24]. However, nothing is known about the underlying cellular functions modified in *E. histolytica*, where NO exposure leads to low ethanol and ATP levels [11]. Analysis of gene expression in trophozoites exposed to NO revealed over-expression of PFOR at the mRNA level. This enzyme is of interest, since it stands at the crossroads between energy production pathways using amino acids and carbohydrates. By taking advantage of available antibodies, we checked the abundance of PFOR in total protein extracts from SNP-treated trophozoites. Whereas *pfor* mRNA was more abundant (a 2.99-fold increase) after treatment with SNP, the protein level did not change (Figure 4), a fact probably due to the massive translational activity reduction during stress or the increased turnover of damaged proteins. These facts prompted us to look at whether a modification in PFOR activity was observed on SNP treatment. As shown in Table 3, a decrease in PFOR activity was observed when virulent parasites were treated with SNP (an 84.7% decrease after treatment for 30 min and an 82.3% decrease after 1 h), despite a higher abundance of *pfor* mRNA. By further incubating parasite extracts in the presence of 1 mM Fe^2+^ under reducing, anaerobic conditions, we show that PFOR low activity was due to irreversible inactivation. To test whether PFOR is a direct target of SNP treatment, PFOR in cytosolic extracts from control amoebae was treated *in vitro* for 20 min with 1 mM SNP in the absence or presence of the NO scavenger PTIO (Table 3). The enzyme was highly inhibited by *in vitro* SNP treatment; however, this inhibitory effect was overcome by PTIO, providing evidencing for a direct inactivation of PFOR by NO species.

**Table 3:** PFOR activities upon no treatment.

I. PFOR activity after *in vivo* treatment with SNP and *in vitro* reactivation				
SNP treatment	**−**	**+**	**+**	**+**
Reactivation	**−**	**−**	1 h	2 h
Activity at 30 min	1.11±0.12	0.17±0.18	0.19±0.14	0.18±0.11
Activity at 1 h	1.30±0.23	0.23±0.19	0.07±0.03	0.08±0.02

II. PFOR activity after *in vitro* incubation with SNP and PTIO				
SNP	**−**	**+**	**+**	
PTIO	**−**	**−**	**+**	
Remaining activity (%)	100	9±7	97.5±2.5	

N = 3 biological replicates.

I. PFOR *in vivo* inactivation and *in vitro* reactivation after treatment of amoebae with SNP. Trophozoites were incubated for 30 or 60 min in culture medium in the absence or presence of SNP. Clarified extracts were prepared under anaerobic conditions and PFOR activity (in U/mg protein) was then determined. PFOR *in vitro* reactivation was performed in extracts of SNP-treated amoebas by incubating with DTT and Fe^2+^ for 1 or 2 h.

II. PFOR *in vitro* inactivation by SNP. Soluble fractions from control amoebae were prepared under anaerobic conditions. Aliquots were incubated in the presence of SNP and in the absence or presence of PTIO for 20 min; the remaining PFOR activity was determined as the ratio of the results obtained for the tested condition and for untreated extracts. Two independent amoeba extracts were assayed, both with control PFOR activities of 1.2 U/mg cellular protein.

In order to further correlate the changes in ER morphology and depletion of ATP with the function of enzymes involved in primary metabolism that showed transcriptional changes, the activities of HK, PGI, GAPDH, PGAM, ENO, PPDK, alcohol dehydrogenases (NADH-dependent ADH, NADPH-dependent ADH), malic enzyme and malate dehydrogenase were also determined in amoeba treated with SNP (Table 4). In addition to the PFOR inhibition described above, significant decreased activities were found for the two types of ADHs and malic enzyme. Malate dehydrogenase activity could not be detected in our kinetic assays.

**Table 4:** Glycolytic and cysteine synthase enzyme activities.

	− SNP	+ SNP
ENZYME	Control enzyme activity	Remaining activity
	(mU/mg cellular protein)	(% of the control)
HK	23	100
PGI	323	100
GAPDH	635	99
PGAM	21–82^a^	89
ENO	296–489^a^	88
PPDK	141	94
NADH-ADH	617–785^a^	26±7
NADPH-ADH	14–66^b^	48±6
Malic enzyme	201–376^b^	66±2
Cysteine synthase	980±110^b^	145±13*

Interval of activities in two (^a^) and four (^b^) independent extracts.

* Two-tailed Student's t-test for non-paired samples, p<0.02 versus control amebas. One unit of enzyme activity is 1 µmol of substrate transformed in 1 min.

Trophozoites were incubated for 1 h in culture medium in the absence or presence of SNP. Clarified extracts were prepared under anaerobic conditions and enzymatic activities were then determined. Remaining activities were determined as the ratio of the results obtained after SNP treatment and for untreated parasites.

To gain insight on our redox findings, the Cys synthase (CS) activity was also determined in amoebas incubated in the absence or presence of SNP. In agreement with the microarray results, CS activity was increased by 45% in trophozoites treated with the stressor although CS activity found was, however, lower than that previously reported for *E. histolytica* (7000 mU/mg protein) [40] which may be due to the modifications made to the enzymatic assay protocol.

Overall this cellular and molecular analysis highlighted that *E. histolytica* has a strong and complex response to NO making a link between ER stress via Hsp activation, redox reactions and glucose metabolism which may account for a decreased ATP level and correlates with the ER morphology changes observed by microscopy.

## Discussion

*Entamoeba histolytica* develops the ability to survive during infection by resisting the host's immunological responses and previous work has suggested that activation of parasitic stress responses occurs during the invasive process [8].

Eukaryotic cells have evolved sophisticated mechanisms to sense stress in intracellular compartments such as the ER and respond appropriately by modulating nuclear gene expression. Within the ER, stress is induced by the presence of large amounts of unfolded or misfolded proteins [4]. Cells can alleviate this shock by degrading or refolding the improperly folded proteins, one activity that needs an overproduction of chaperones.

As in a previous study [25], we identified an upregulation of *hsp* genes. These were here newly annotated using a desktop application we developed for the retrieval of annotations of *E. histolytica*'s genome, thus providing the community with a genuine tool for rapid analysis of functional genomic data. We identified additional genes that were upregulated (*hsp90*), while commonly identified genes (*hsp20*, *−70* and −*101*) exhibited greater fold changes in our study. A more intense shock was induced in our experiments, due to the higher concentration of the NO donor used (1 mM versus 200 µM) and may explain the fact that only our dataset provide identification of a large panel of HSPs, whole pathways for DNA repair, metabolism or protein degradation.

The stress response triggers the induction of *hsp* gene transcription upon exposure of cells to proteotoxic environments, which is a mechanism conserved from bacteria to mammals. In extreme stress, the protein quality control system can become saturated; this leads to accumulation and aggregation of misfolded proteins [26], [27]. These aggregates can potentially be eliminated by proteasome-mediated proteolysis in the cytosol. Under high thermal stress conditions (*e.g.* >50°C), cells also induce a thermotolerance process capable of reactivating aggregated proteins *via* the bi-chaperone Hsp101 (ClpB)-Hsp70 system, whose genes are seen to be the most highly over-expressed in *E. histolytica* in the presence of NO.

In our experiments, *hsp* genes were overexpressed and massive cell death occurred. The protective function of *hsp* genes may be overwhelmed by misfolded proteins, resulting in cell death by loss of protein function or toxicity, however, one cannot exclude that prolonged hsp gene overexpression might be detrimental to cells.

Following SNP internalization and its activation, the NO generated can interact with a variety of molecules, especially aromatic residues, amines, thiols and metal ion-containing centers (such as heme or Fe-S clusters) and causes an inhibition of enzyme active sites. These assumptions suggest that increased turnover of Fe-S cluster-containing proteins and/or augmented Fe-S cluster synthesis or reparation is required to overcome the deleterious effects of NO treatment. Remarkably, we observed over-expression of genes encoding enzymes responsible for Fe-S cluster genesis and CS, the latter involved in the synthesis of Cys, the main antioxidant metabolite in amoeba and an essential aminoacid constituent of Fe-S clusters. The increased CS mRNA correlated with significant higher CS activity in the amebas exposed to SNP compared to control parasites. We also observed overexpression of Fe-S cluster-containing proteins such as PFOR, a metabolic enzyme that is essential in glucose metabolism in the parasite [24]. Active PFOR requires intact Fe-S clusters and their transfer onto native PFOR apoproteins. The oxido-reductive properties of the iron atom make it highly susceptible to attack by ROS [24]. The present results indicated that NO also inhibited the enzyme, but unlike to the reversible inactivation by ROS, NO produced a pronounced and irreversible inactivation. As fixation of Fe-S clusters by PFOR is mediated by cysteine residues, the inactivation we observed could be due to the formation of adducts between NO and the sulphur atoms of these amino acids. In the experiments we performed, either RNAs or proteins were purified directly after incubation with SNP; this implies that newly synthesized and active PFOR enzymes would be deactivated, as we have shown *in vitro* by incubating active enzymes with SNP. Similar results were obtained for other glycolysis-related enzymes: a 4.3-fold up-regulation was observed for the gene that encodes the bifunctional aldehyde-alcohol dehydrogenase 2, the main NADH-dependent alcohol dehydrogenase activity in the parasite responsible of ethanol production and NADH reoxidation under anaerobic/microaerophilic conditions which was severely inhibited by SNP treatment and which has a prominent role in determining the glucose catabolism end-product profile in the parasites [24]. Moreover, the NADPH-dependent ADH activity (*alcohol dehydrogenase 3* gene) was also inhibited, although to a lesser extent; however, its contribution to ethanol synthesis is not well established yet. Malic enzyme activity was also inhibited by SNP treatment, which, together with malate dehydrogenase, is involved in reoxidation of the NADH produced during glycolysis under partial aerobic conditions where ADH2 is inhibited.

These results highlight that energy metabolism is highly impaired by NO, leading to a decrease in the levels of their end-products – ethanol and ATP [11] – and maybe in the redox potential. These results also correlate with high glucose-6-phosphate (G6P), fructose-6-phosphate (F6P) and dihydroxyacetone-phosphate (DHAP) concentrations in NO-treated trophozoites [11]; these accumulated metabolites are intermediates of reactions upstream of PFOR in the glycolytic pathway. Thus, our results indicate that the glycolysis-fermentation pathway is disrupted upon NO treatment and that a feedback response may lead to enhanced transcription of *pfor* and other genes involved in glucose metabolism.

Cells will promote their survival by reducing misfolded protein levels during stress by activating UPR. Unexpectedly, the *E. histolytica* genome does not contain gene paralogues encoding important UPR-related proteins such as PERK or ATF6 and indeed *E. histolytica* does not seem to induce UPR upon NO treatment. The absence of UPR responses (or defects herein) has been found in other protozoan [28] and has been noticed as well in important diseases such as in the Wolfram syndrome which includes diabetes at early stages, mutations in WFSI (Wolframine) leads to high levels of ER stress and cell apoptosis. WFS1 is a ER transmembrane protein acting in calcium homeostasis and it has recently been shown that WFS1 is a key feedback regulator of the ATF6 branch of the UPR [29]. These evidences suggest that ER stress is a major player in the establishment of important human pathologies such as diabetes, characterized by high blood glucose levels, contributing to pancreatic ß-cell death and insulin resistance. Interestingly, *in vitro* data suggest that the cytokines IL-1β and IFN-γ, putative mediators of ß -cell loss in type-1 diabetes, induce severe ER stress through NO-mediated depletion of ER calcium and inhibition of ER chaperones, respectively, thus hampering ß -cell defenses and amplifying the pro-apoptotic pathways. The physiopathology of ß -cells during type-1 diabetes thus share common features with *E. histolytica's* response to NO.

One interesting aspect on ER morphology changes is that the ER components remains localized to the same compartments during the ER fission process. This fact indicates that the parasite has developed a way for the ER not to be obliterated upon contact with stress components. ER fragmentation and the subsequent potential and/or eventual fusion may be one of *E. histolytica's* strategies to rapidly recover ER function after removal of a stress, allowing its survival.

Reversible ER fission is possible and has been evidenced in neurons, depending on mechanisms other than energy depletion and cell death, whereas extracellular Ca^2+^ is obligatory for ER fragmentation [30]. This other cellular system suggests that ER fission is not solely a passive mechanism due to a lack of energy, but may also be a way for the cell to respond to a stress, probably by compartmentalizing the continuous ER to avoid diffusion of toxic products within the organelle and towards the nucleus.

The cellular and molecular analysis presented here has highlighted a strong and complex response to NO linked to ER stress and glucose metabolism which is a topic of interest to understand the death of *E. histolytica* during amoebiasis and also the death of other cells during human metabolic diseases.

## Materials and Methods

### *Entamoeba histolytica* culture

*E. histolytica* trophozoites (strain HM1:IMSS) were cultured in TYI-S-33 medium at 37°C [31]. Vector or GFP-KDEL (Kindly provided by C. Huston, Vermont) transfected parasites were maintained in 10 µg G418 and were cultured in the presence of 30 µg G418 for 2 days before each experiment.

### Treatment of parasites with nitric oxide

Trophozoites (5×10^5^) were incubated at 37°C in 13 ml culture medium with/without 1 mM sodium nitroprusside (SNP; #431451, Aldrich). The survival of treated trophozoites was equal to 50.8%±4.3% (n = 2), in agreement with previous results [11]. Three independent experiments were performed, averaged and confidence interval (α = 0.05) was calculated (n = 3). Alternatively, standard error was calculated and results were compared using two-tailed t-test.

### Live parasites imaging

To restrict amoeba detachment we used a type I bovine collagen matrix. Collagen matrix was created by mixing 1.7 mg/mL bovine collagen type I (BD Biosciences) with incomplete (without serum) amoeba media (TYI), 8.3 mM NaOH, and 90,000 amoebas expressing GFP-KDEL-FLAG. After mixing, 100 µl of the matrix was added to each imaging dish (MatTek Corporation, USA) and then incubated at 37°C for 30 min to polymerize. Immediately before imaging either a coverslip was added directly to the top of the matrix for untreated samples or 1 mM SNP was added to the top of the matrix before applying the coverslip. The amoebas were then imaged using the Spinning Disc Confocal Microscope (Perkin Elmer, USA). All images were acquired using the 40× objective and taking one stack every minute for 1 h using a 0.5 µm Z-step. After acquisition the images were analyzed using Imaris (Bitplane Scientific Software, St. Paul, MN, USA) and Image J (http://rsb.info.nih.gov/ij/) softwares. For the Imaris analysis, the out of focus slices were removed, a 0.355 µm Gaussian filter was applied and 3D reconstructions were generated. Time points were chosen for the analysis (0, 25, and 45 min).

### Immunofluorescence and confocal microscopy analysis

Trophozoites were incubated at 37°C in 13 ml culture medium with/without 1 mM SNP for 1 h. The parasites were then centrifuged for 5 min at 550× *g*; the pellet was fixed in 4% paraformaldehyde at 37°C for 15 min. After, the fixed trophozoites were treated for confocal microscopy as published (15). Primary antibodies used were anti-FLAG M2 (1∶500) (Sigma), anti-CRT (1∶500) [32]. Images were acquired using the Spinning Disc Confocal Microscope (Perkin Elmer, USA) equipped with the 63× objective with slices taken at 0.5 µm steps for an average of 30 slices per amoeba. Three-dimensional representations of confocal image stacks were generated using the software package Imaris (Bitplane Scientific Software, St. Paul, MN, USA). After the confocal image stack was loaded into Imaris, a “surface object” was created for each channel to generate the 3-D model. The intensity threshold for each surface object was adjusted to best match the original image. For each image a 3D reconstruction was generated by applying the following analysis tools: surfaces area detail level of 1 µm, a background threshold of 3.75 µm, an automatic threshold background subtraction, and a filter to include all staining areas with a volume greater than 10 µm^3^.

### RNA purification and microarray analysis

Ten million trophozoites were incubated for 1 h at 37°C in 250 ml culture medium in the presence or absence of 1 mM SNP. Parasites were then recovered by centrifugation (550× *g*) and RNA purification was carried out as previously described [8]. We designed new microarrays (EH-IP2008; Agilent Technologies) to cover the entire *E. histolytica* ORFs using data gathered from our cDNA library sequencing [12], Pathema (2008, http://pathema.jcvi.org/cgi-bin/Entamoeba/PathemaHomePage.cgi) and TIGR databases (2005, http://www.tigr.org/tdb/e2k1/eha1/eha1.shtml). In total, 30,274 unique probes, with an average Tm of 79°C±1.5°C and sizes ranging between 45 and 60 nt, – were designed and covered 11,622 predicted ORFs, with an average density of 2.6 probes/ORF. Specificity and nature of probes are found in the genoscript open access data base, whose accession link is cited below. Microarrays experiments were performed as published [8]. For each version of the chip, two biological replicates were performed, each of them having two technical replicates hybridized in dye swap (thus yielding 8 hybridized slides). Statistical analyses were carried out with the R software (http://www.R-project.org) and Bioconductor packages (http://www.bioconductor.org). A lowess normalization was first performed on all spots using the normalizeWithinArrays function of the limma package [33]. The moderated t-test included in the limma [34] package was finally applied on each gene and raw *p* values were adjusted for multiple testing by the Benjamini and Yekutieli (BY) method [35], which controls the false discovery rate (FDR). We considered genes as being differentially expressed when the BY *p* value≤0.05. Only genes with an expression fold change (FC) ≥2 were kept in the present analysis (Tables S1 and S2). Nevertheless, complete experimental details and data sets are available online at our “genoscript” MIAME-based data library(http://genoscript.pasteur.fr) with the accession link: http://genoscript.pasteur.fr/cgibin/WebObjects/GenoScript.woa/1/wa/ListDirectAction/inspectDap?pkentity=142

### Interpretation of microarray results

Results obtained with the two sets of microarrays were classified by gene, according to the available genome annotation (GenBank accession: AAFB00000000.2). In cases when several probes targeted a single gene, the base 2 logarithms of their expression fold change (“Log_2_ ratio”) were averaged. The correlation between the two datasets was then evaluated by Pearson's coefficient. To explore the data we developed a desktop application to host the functional annotations of the proteins along with our microarray data, enabling rapid retrieval of the annotations of the differentially expressed genes. These annotations include BLAST hits against GenBank RefSeq, KEGG pathways and Gene Ontologies annotated using BLAST2GO [36] conserved domains annotated using InterProScan (www.ebi.ac.uk/Tools/pfa/iprscan/), as well as protein families annotated using spectral clustering [37]. This application was developed under the Cocoa frameworks of Apple Inc. The application is free and available from the authors upon request. Finally, GO Slim Terms implemented in AmiGO (http://amigo.geneontology.org/cgi-bin/amigo/slimmer), based on the GO annotations in AmoebaDB version 1.3.

### Protein analysis by immunoblotting

Trophozoite protein extracts were obtained after 1 h treatment in the presence or absence of 1 mM SNP followed by immunoblotting (proteins from up to 80,000 cells were loaded per lane). The primary antibody used were a rabbit anti-PFOR polyclonal antibody (1∶400 dilution, a kind gift from Dr Ester Orozco, CINVESTAV, Mexico), a mouse anti-actin monoclonal antibody (1∶20,000 dilution; #69100, MP Biomedicals). Scanned autoradiographs were analyzed using the ImageJ software and protein abundance normalized with actin values. The primary antibodies used were a rabbit anti-CRT polyclonal antibody (1∶2,000 dilution [32]) and an anti-α-actinin2 polyclonal antibody (1∶4,000 dilution) generated by immunizing rabbits with two peptides form EHI_199000 ORF (NETKNYRKGDKRAFI and YMKEKNDENPSPEQL). These antibodies were manufactured by Eurogentec (France).

### Pyruvate:ferredoxin oxidoreductase assays

PFOR activity was determined under anaerobic conditions as previously described [24]. For *in vivo* PFOR inactivation, 20 million trophozoites separated in individual tubes at a density of 1×10^6^/ml of TYI-S-33 medium were incubated in the absence or presence of 1 mM SNP for 1 h at 37°C. The samples were pooled, harvested and washed once with PBS; the pellets were resuspended in 0.5 ml of 100 mM KH_2_PO_4_ pH 7.5 buffer (previously purged with N_2_) supplemented with 25 mM β-mercaptoethanol, 1 mM PMSF, 5 mM EDTA and 1% Triton X-100. The cells were lysed by freeze/thaw cycles in liquid nitrogen and centrifuged at 21,000× g; the supernatant was then recovered and stored under anaerobic conditions for PFOR activity determination. For enzyme reactivation from amoeba treated with SNP, aliquots of the latter extracts were treated under anaerobic conditions for 1 or 2 h with 1 mM Fe^2+^ and 5 mM DTT; the PFOR activity was then determined. For PFOR *in vitro* inactivation, anaerobic cytosolic extracts from control trophozoites were prepared and 50 µl (0.5–0.55 mg) were incubated for 20 min with 1 mM SNP on ice in a sealed 0.2 ml tube. In parallel, aliquots in the presence of 1 mM SNP and 1 mM of the NO scavenger PTIO (2-phenyl-4,4,5,5-tetramethylimidazoline-1oxyl-3-oxide; SIGMA) were also prepared and the remaining PFOR activity was determined.

### Glycolytic enzyme and cysteine synthase assays

One hundred million trophozoites previously harvested and washed twice with PBS were incubated at a density of 4–8×10^6^/ml in TYI-S33 medium in the absence or presence of 1 mM SNP for 1 h at 37°C. The cells were further harvested and washed once with PBS and cytosolic extracts were obtained as previously described [38]. Glycolytic enzyme activities for hexokinase (HK), phosphoglucose isomerase (PGI), glyceraldehyde-3-phosphate dehydrogenase (GAPDH), 2,3-bisphosphoglycerate-independent 3-phosphoglycerate mutase (PGAM), enolase (ENO) and pyruvate phosphate dikinase (PPDK) were determined as previously described [39]. The enzymatic assays for NAD^+^ and NADP^+^ ADHs were performed in 100 mM pyrophosphate-phosphoric acid buffer pH 8.8 purged with N_2_, in which were added 10 mM freshly prepared cysteine, 2 mM NAD^+^ or 1 mM NADP^+^ depending on the enzyme, freshly prepared cytosolic extract (0.05–0.15 mg for NAD^+^ ADH; 0.1–0.5 mg for NADP^+^ ADH); the reaction was started by adding 170 mM absolute ethanol. Malic enzyme assay consisted of a mixture of buffers at pH 7.0 (50 mM imidazole, 10 mM each of acetate, MES and Tris), 5 mM MgCl_2_, 1 mM NADP^+^, 0.05–0.1 mg cytosolic extract and the reaction started by adding 10 mM malate. Malate dehydrogenase activity was measured in a similar assay to that of malic enzyme, but 0.2 mM NADH replaced NADP^+^ and the reaction was started by adding 0.5 mM oxaloacetate. Care was taken that the enzyme rates were linear in the interval of the proteins tested and the baseline absorbance in the absence of the specific substrate was always subtracted. All the kinetic assays were determined at a temperature of 37°C; NAD(P)^+^ reduction or NADH oxidation were monitored at 340 nm in a spectrophotometer (Agilent, Sta. Clara, CA). One unit of enzymatic activity is 1 µmol.min^-1^.

Cysteine synthase activity was determined by derivatizing with ninhydrin as previously described [40] with the following modifications: Cytosolic extracts were incubated in 50 mM Tris-HCl pH 7.5, 2 mM Na_2_S and 2 mM O-acetyl serine (OAS) in a volume of 100 microliters. A reaction without OAS was run in parallel to account for the spurious reaction of ninhydrin with free amino acids and proteins. After 10 min incubation at 37°C, the samples were processed as described in [41]. The high absorbance value of the reaction without the specific substrate was subtracted to that of the complete reaction. The nmoles of synthesized Cys were determined as described in [41] from a standard curve of 0–40 nmoles Cys using a solution previously calibrated with 5,5′- dithiobis (2-nitrobenzoic acid) (Σ_412 nm_ = 13600 M^−1^ cm^−1^). The Cys value was corroborated using the extinction coefficient of Σ_560 nm_ = 26×10^3^ M^−1^ cm^−1^ reported for the Cys-ninhydrin reagent 2 complex [41]. It was taken care that the activity was linear with respect to the protein concentration (0.5–2 µg of clarified extract) and to the incubation time (max. 10 min). Outside of these intervals, the reaction exceeds the limits of confidence of the assay.

## Supporting Information

Figure S1 **Correlation between the results obtained with EH-IP2007 and EH-IP2008 microarrays.** An empty circle represents each gene targeted by a probe in both versions of the chip. The log_2_ ratios of gene expression modulation in response to NO are plotted as indicated in the figure. The red line represents the equality of the results between the two microarrays. The Pearson's correlation coefficient (R^2^ = 0.85) revealed a positive linear relationship between the results obtained with both chip versions.
(TIF)

Video S1 **ER morphology in** ***E. histolytica.*** Trophozoites expressing GFP-KDEL-FLAG were seeded on glass bottom culture dishes (MatTeck) embedded in a collagen matrix and incubated at 37°C. Immediately before imaging a coverslip was added directly to the top of the matrix. The amoebas were then imaged using the Perkin Elmer Spinning Disc Confocal Microscope. All images were acquired using the 40× objective and taking one stack every minute for 1 h using a 0.5 µm Z-step.
(AVI)

Video S2 **ER morphology in** ***E. histolytica*** **treated by nitric oxide.** Trophozoites expressing GFP-KDEL-FLAG were seeded on glass bottom culture dishes (MatTeck) embedded in a collagen matrix and incubated at 37°C. Immediately before imaging 1 mM SNP was added directly to the top of the matrix that then was covered by a coverslip. The amoebas were then imaged using the Perkin Elmer Spinning Disc Confocal Microscope. All images were acquired using the 40× objective and taking one stack every minute for 1 h using a 0.5 µm Z-step.
(AVI)

Table S1 **Genes upregulated by NO treatment.**
(PDF)

Table S2 **Genes downregulated by NO treatment.**
(PDF)

Table S3 **GOSlim Terms of Genes modulated by NO treatment.** The data were analyzed using GO Slim Terms implemented in AmiGO (http://amigo.geneontology.org/cgi-bin/amigo/slimmer), based on the GO annotations in AmoebaDB version 1.3. (http://amoebadb.org/amoeba/).
(PDF)

Table S4 **Genes modulated by treatment with nitric oxide.**
(PDF)
