# Supplementary figures and images for: Endoplasmic Reticulum Stress-Sensing Mechanism Is Activated in Entamoeba histolytica upon Treatment with Nitric Oxide
Source: PLoS One. 2012 Feb 24;7(2):e31777. doi: 10.1371/journal.pone.0031777 (PMC3286455; doi:10.1371/journal.pone.0031777)

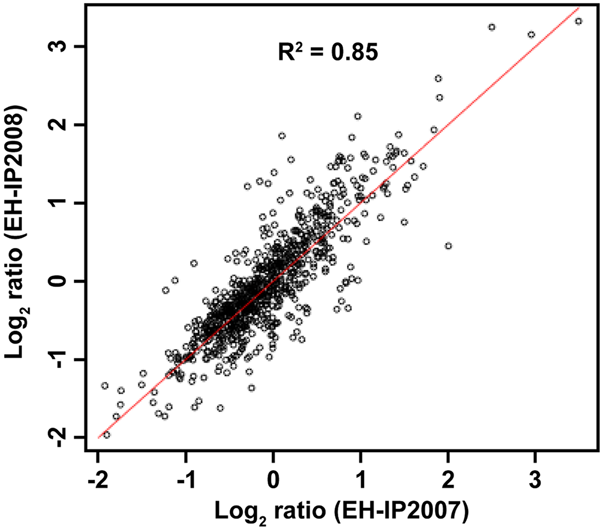

Supplement: Figure S1 — Correlation between the results obtained with EH-IP2007 and EH-IP2008 microarrays. An empty circle represents each gene targeted by a probe in both versions of the chip. The log2 ratios of gene expression modulation in response to NO are plotted as indicated in the figure. The red line represents the equality of the results between the two microarrays. The Pearson's correlation coefficient (R2 = 0.85) revealed a positive linear relationship between the results obtained with both chip versions. (TIF) [file pone.0031777.s001.tif]
