# Supplementary material for: Endoplasmic Reticulum Stress-Sensing Mechanism Is Activated in Entamoeba histolytica upon Treatment with Nitric Oxide
Source: PLoS One. 2012 Feb 24;7(2):e31777. doi: 10.1371/journal.pone.0031777 (PMC3286455; doi:10.1371/journal.pone.0031777)
Supplement: Table S1 — Genes upregulated by NO treatment. (PDF) [file pone.0031777.s004.pdf]

Table S1. Genes upregulated by NO treatment				
**GENE ID**	**Description**	**FC**	**BY**	**rawp**
64.t00033	heat shock protein 101, putative	66,6	2E-04	7E-08
EHI_156560	heat shock protein, putative	59,7	2E-04	6E-08
82.t00010	AAA family ATPase, putative	55,8	2E-04	8E-08
EHI_183680	heat shock protein 101, putative	50,0	1E-04	2E-08
64.t00034	heat shock protein 101, putative	43,5	3E-04	7E-07
525.t00001	heat shock protein 101, putative	42,0	2E-04	7E-08
EHI_155060	chaperone clpB, putative	37,3	2E-04	1E-07
511.t00001	HSP101-related protein	36,4	2E-04	5E-07
EHI_076480	heat shock protein 101, putative	36,4	2E-04	1E-07
EHI_094680	chaperone clpB, putative	36,3	2E-04	3E-07
EHI_061640	heat shock protein70, hsp70A2, putative	33,7	7E-04	8E-06
81.t00001	heat shock protein 101, putative	32,4	2E-04	9E-08
350.t00001	heat shock protein 101, putative	30,0	2E-04	2E-07
EHI_132540	heat shock protein70, hsp70A2, putative	29,3	5E-04	5E-06
EHI_094470	heat shock protein 101, putative	27,2	2E-04	2E-07
EHI_013550	heat shock protein 101, putative	26,5	3E-04	7E-07
EHI_178230	heat shock protein 101, putative	25,3	2E-04	2E-07
EHI_090840	chaperone clpB, putative	24,7	6E-04	6E-06
301.t00005	heat shock protein 70, putative	23,4	5E-04	4E-06
EHI_192440	heat shock protein70, hsp70A2, putative	22,1	8E-04	1E-05
EHI_132530	heat shock protein70, hsp70A2, putative	21,8	6E-04	7E-06
181.t00013	hsp101-related protein	19,4	2E-04	4E-07
872.t00001	heat shock protein 101, putative	17,7	2E-04	3E-07
EHI_015390	heat shock protein70, hsp70A2, putative	16,6	4E-04	3E-06
EHI_108130	heat shock protein70, hsp70A2, putative	16,0	6E-04	6E-06
EHI_055680	heat shock protein, Hsp20 family, putative	15,9	5E-04	4E-06
EHI_026590	heat shock protein 70, putative	14,9	6E-04	6E-06
52.t00022	hsp70 family protein	11,7	6E-04	8E-06
284.t00002	heat shock protein 90, putative	10,1	4E-05	1E-09
EHI_197860	hsp70 family protein	10,0	2E-04	3E-07
92.t00037	heat shock protein 90, putative	9,9	4E-05	1E-09
EHI_187000	DnaJ family protein	9,6	5E-04	5E-06
EHI_196940	heat shock protein 90, putative	7,9	6E-05	4E-09
EHI_138480	iron-sulfur flavoprotein, putative	7,3	2E-04	5E-07
EHI_052860	heat shock protein 70, putative	6,7	1E-04	4E-08
361.t00007	ubiquitin-activating enzyme, putative	6,6	2E-04	8E-08
EHI_151260	DnaJ family protein	6,3	2E-04	7E-08
EHI_056490	20 kDa antigen-related protein	6,2	7E-05	7E-09
EHI_185410	26S protease regulatory subunit, putative	6,1	3E-04	1E-06
EHI_145720	Rab family GTPase	6,1	4E-04	3E-06
EHI_157010	alcohol dehydrogenase, putative	5,9	6E-05	4E-09
49.t00025	ubiquitin-activating enzyme, putative	5,9	1E-04	3E-08
EHI_107560	alcohol dehydrogenase, putative	5,9	6E-05	5E-09
EHI_023300	TPR repeat protein	5,5	2E-04	2E-07
EHI_178850	70 kDa peptidyl-prolyl isomerase, putative	5,4	2E-04	3E-07
155.t00018	inositol polyphosphate-5-phosphatase, putative	5,4	4E-04	2E-06
EHI_039190	aldose reductase, putative	5,4	2E-04	4E-08
EHI_105080	zinc finger protein, putative	5,4	1E-04	3E-08
EHI_026480	(2r)-phospho-3-sulfolactate synthase, putative	5,3	2E-04	3E-07
EHI_020270	ubiquitin-activating enzyme, putative	5,3	3E-04	1E-06
23.t00028	glutamyl-tRNA synthetase, putative	5,2	9E-05	1E-08
153.t00022	predicted protein	5,2	4E-04	2E-06
EHI_045120	cell division cycle protein 48, putative	5,2	3E-04	1E-06
EHI_093330	Helix-turn-helix protein, putative	5,2	9E-05	1E-08
328.t00005	cysteine synthase	5,2	2E-04	8E-08
EHI_160570	26S protease regulatory subunit 8, putative	5,2	2E-04	4E-07
EHI_155570	glutamyl-tRNA synthetase, putative (gltX) [6.1.1.17]	5,1	1E-04	3E-08
EHI_176970	cdc48-like protein, putative	5,0	2E-04	4E-07
84.t00012	peptidyl-prolyl cis-trans isomerase, putative	5,0	2E-04	4E-07
EHI_194070	26S protease regulatory subunit S10B, putative	4,7	3E-04	6E-07
EHI_183120	saliva IgA-recognized antigen-related	4,7	2E-04	1E-07
207.t00001	calponin homology domain protein, putative	4,7	4E-04	2E-06
EHI_133220	myb-like DNA-binding domain containing protein	4,6	3E-04	1E-06
507.t00004	26s protease regulatory subunit	4,5	2E-04	5E-07
EHI_160930	Cysteine synthase A, putative (cysK) [4.2.99.8]	4,5	2E-04	5E-07
16.t00071	acyl-CoA synthetase, putative	4,3	9E-05	1E-08
33.t00021	cysteine desulfurase, putative	4,3	3E-04	7E-07
EHI_187600	26S protease regulatory subunit, putative	4,3	5E-04	6E-06
EHI_025710	iron-sulfur flavoprotein, putative	4,2	3E-04	6E-07
EHI_178580	ATP-binding cassette protein, putative	4,2	9E-05	1E-08

45.t00023	protein with DnaJ and myb domains	4,1	5E-04	3E-06
EHI_150390	aspartate ammonia-lyase, putative	4,1	2E-04	1E-07
105.t00005	ACC protein, putative	4,1	2E-04	2E-07
275.t00011	protein kinase, putative	4,1	3E-04	1E-06
EHI_013180	dynamin-1-like protein, putative	4,0	4E-04	2E-06
EHI_171750	cysteine synthase 2, putative	4,0	6E-04	7E-06
EHI_024240	aldehyde-alcohol dehydrogenase 2, putative	4,0	3E-04	1E-06
EHI_053020	26S protease regulatory subunit, putative (proteinforMGC:5)	4,0	4E-04	3E-06
EHI_193390	heat shock protein, Hsp20 family, putative	3,9	2E-04	2E-07
328.t00012	NAD(FAD)-dependent dehydrogenase, putative	3,9	3E-04	1E-06
EHI_023090	recQ family DNA helicase	3,9	2E-04	2E-07
36.t00036	Truncated ABC transporter, putative	3,9	2E-04	4E-08
EHI_122710	TPR repeat protein	3,9	4E-04	3E-06
EHI_158050	Hsc70-interacting protein, putative	3,9	2E-04	3E-07
9.t00085	RNA-binding protein, putative	3,9	1E-04	3E-08
EHI_055630	ATP-binding cassette, sub-family C, putative	3,8	1E-04	2E-08
EHI_090640	cylicin-2, putative	3,8	3E-04	1E-06
4.t00018	26S proteasome regulatory subunit, putative	3,8	2E-04	1E-07
26.t00026	RNA-binding protein, putative	3,8	2E-04	4E-08
22.t00032	aspartase-related, putative	3,7	2E-04	7E-08
EHI_112990	2,3-bisphosphoglycerate-independentphosphoglycerate mutase, putati	3,7	3E-04	1E-06
EHI_082270	aspartate ammonia-lyase, putative	3,6	3E-04	8E-07
14.t00035	dynamin-ike protein, putative	3,6	3E-04	9E-07
EHI_096770	acetyltransferase, putative	3,6	2E-04	8E-08
EHI_128200	protein with DnaJ and myb domains	3,6	2E-04	1E-07
EHI_050830	DNA-directed RNA polymerase III subunit, putative	3,6	2E-04	3E-07
328.t00011	glycerol-3-phosphate dehydrogenase, putative	3,5	5E-04	5E-06
EHI_099700	NAD(FAD)-dependent dehydrogenase, putative	3,5	3E-04	7E-07
173.t00005	NAD(FAD)-dependent dehydrogenase, putative	3,5	4E-04	2E-06
487.t00001	NAD+ dependent alcohol dehydrogenase-related	3,5	5E-04	5E-06
EHI_023890	nuclear movement protein, putative	3,4	2E-04	4E-07
EHI_134340	glutamic acid-rich protein precursor, putative	3,4	1E-03	2E-05
EHI_185240	long-chain-fatty-acid--CoA ligase, putative	3,4	2E-04	5E-08
EHI_060340	Cysteine synthase A, putative (cysK) [4.2.99.8]	3,4	6E-04	6E-06
EHI_084730	multidrug resistance-associated protein, putative	3,4	2E-04	7E-08
40.t00026	DNA replication licensing factor, putative	3,3	3E-04	1E-06
EHI_194570	26S protease regulatory subunit, putative	3,3	5E-04	4E-06
EHI_012490	ARF GTPase activating protein, putative	3,3	3E-04	6E-07
EHI_136380	cysteine desulfurase, putative (nifSproteinhomo)	3,3	6E-04	6E-06
EHI_078740	Rho guanine nucleotide exchange factor, putative	3,3	2E-04	9E-08
EHI_187180	ubiquitin-protein ligase, putative	3,3	4E-04	3E-06
EHI_094260	exonuclease I, putative	3,3	2E-04	7E-08
EHI_029600	leucine rich repeat protein	3,3	2E-04	2E-07
EHI_054240	DNA repair helicase, putative	3,2	3E-04	9E-07
EHI_150490	alcohol dehydrogenase, putative (adh2)	3,2	4E-04	2E-06
EHI_088070	ribosomal RNA methyltransferase, putative	3,2	3E-04	2E-06
282.t00012	dynamin-like protein	3,1	3E-04	7E-07
EHI_131670	dynamin-1-like protein, putative	3,1	5E-04	5E-06
351.t00001	phosphoglycerate mutase, 2,3-bisphosphoglycerate-independent, puta	3,1	4E-04	2E-06
EHI_155490	heat shock protein 70, putative	3,1	4E-04	2E-06
EHI_137730	ubiquitin carboxyl-terminal hydrolase, putative	3,1	2E-03	5E-05
454.t00003	CXXC-rich protein	3,0	3E-04	1E-06
EHI_152430	cell division protein kinase, putative	3,0	3E-04	9E-07
EHI_160940	aldehyde-alcohol dehydrogenase 2	3,0	4E-04	2E-06
EHI_009530	pyruvate phosphate dikinase (ppdK) [2.7.9.1]	3,0	6E-04	6E-06
EHI_166050	mitochondrial-typeheat shock protein70, putative	3,0	3E-04	8E-07
21.t00005	26S proteasome regulatory complex subunit	3,0	2E-04	1E-07
EHI_155290	actinin-like protein, putative	3,0	2E-04	4E-07
EHI_035920	regulator of nonsense transcripts, putative	3,0	6E-04	8E-06
EHI_100810	heat shock protein 70, mitochondrial, putative	2,9	3E-04	9E-07
EHI_039180	acetyltransferase, putative	2,9	2E-04	5E-07
34.t00053	alcohol dehydrogenase 3	2,9	3E-04	1E-06
EHI_110520	glutamate synthase small subunit, putative	2,9	2E-04	3E-07
EHI_161010	anaerobic glycerol-3-phosphate dehydrogenase subunit A, putative	2,9	2E-04	4E-07
EHI_044930	eukaryotic translation initiation factor 4 gamma, putative	2,9	2E-04	4E-07
15.t00024	chaperonin-containing TCP-1, zeta subunit	2,9	5E-04	4E-06
EHI_031220	DNA repair protein RAD51, putative	2,9	4E-04	2E-06
EHI_125830	heat shock protein, Hsp20 family	2,9	6E-04	6E-06
EHI_086140	Ras guanine nucleotide exchange factor, putative	2,9	5E-04	5E-06
EHI_125650	PIWI, putative	2,9	5E-04	3E-06
EHI_180350	26S protease regulatory subunit, putative	2,9	6E-04	6E-06
EHI_118180	cullin, putative	2,9	2E-04	2E-07

EHI_177320	26s proteasome subunit P45 family protein, putative	2,9	3E-04	1E-06
EHI_136960	poly(ADP-ribose) polymerase, putative	2,9	2E-04	2E-07
41.t00041	iron-sulfur flavoprotein, putative	2,9	2E-04	5E-07
EHI_092500	RNA recognition motif domain containing protein	2,8	3E-04	1E-06
EHI_178710	phosphoesterase, putative	2,8	5E-04	5E-06
57.t00003	mitochondrial type hsp 70, putative	2,8	2E-04	3E-07
328.t00004	aldehyde-alcohol dehydrogenase 2, putative	2,8	4E-04	2E-06
EHI_125800	chaperonin-containing TCP-1, zeta subunit, putative	2,8	7E-04	9E-06
251.t00015	26S proteasome subunit P45 family protein, putative	2,8	3E-04	1E-06
EHI_051060	pyruvate:ferredoxin oxidoreductase	2,8	9E-04	1E-05
EHI_040060	cylicin-2, putative	2,8	8E-04	1E-05
EHI_120640	DNA topoisomerase II, putative	2,8	3E-04	7E-07
3.t00082	pyruvate phosphate dikinase	2,8	4E-04	2E-06
308.t00007	mitochondrial type hsp 70, putative	2,8	2E-04	4E-07
EHI_009530	pyruvate phosphate dikinase	2,8	5E-04	4E-06
EHI_050940	2,3-bisphosphoglycerate-independent phosphoglycerate mutase), puta	2,8	5E-04	4E-06
EHI_159580	protein kinase, putative	2,8	5E-04	4E-06
EHI_122100	acetyltransferase, putative	2,8	3E-04	8E-07
98.t00023	pyruvate:ferredoxin oxidoreductase	2,8	5E-04	5E-06
57.t00001	mitochondrial type hsp 70, putative	2,7	3E-04	9E-07
47.t00022	cell division control protein 7, putative	2,7	2E-04	1E-07
EHI_164460	leucine-rich repeat containing protein	2,7	2E-04	2E-07
EHI_123840	tyrosine kinase, putative	2,7	3E-04	1E-06
EHI_150110	metallo-beta-lactamase family protein	2,7	4E-04	2E-06
EHI_169630	DEAD/DEAH box helicase, putative	2,7	7E-04	1E-05
EHI_153770	phosphatidylinositol-4-phosphate 5-kinase, putative	2,7	6E-04	8E-06
EHI_155530	chromosome partition protein, putative	2,7	3E-04	2E-06
EHI_076880	DNA replication licensing factor, putative	2,7	2E-04	4E-07
EHI_038690	ubiquitin-activating enzyme E1 1, putative	2,7	8E-04	1E-05
EHI_012220	protein kinase domain containing protein	2,7	3E-04	2E-06
73.t00014	acetyltransferase, putative	2,7	3E-04	7E-07
EHI_153840	midasin, putative	2,6	2E-04	2E-07
EHI_148430	polynucleotide kinase-3'-phosphatase, putative	2,6	2E-03	4E-05
EHI_050760	mutT/nudix family protein	2,6	2E-04	2E-07
244.t00017	predicted protein	2,6	3E-04	9E-07
EHI_154150	suppressor protein SRP40, putative	2,6	1E-03	3E-05
187.t00004	elongation factor 2, putative	2,6	5E-04	4E-06
EHI_131230	actin, putative	2,6	2E-04	3E-07
EHI_045080	pyruvate, phosphate dikinase, putative	2,6	2E-04	4E-07
EHI_055660	peptidase, C54 family	2,6	5E-03	2E-04
EHI_111990	CXXC-rich protein	2,6	5E-04	4E-06
EHI_098550	ubiquitin-activating enzyme, putative	2,6	2E-04	2E-07
11.t00054	chaperonin-containing TCP-1, zeta subunit, putative	2,6	8E-04	1E-05
EHI_112790	acetyltransferase, putative	2,6	4E-04	3E-06
EHI_021500	ankyrin repeat protein, putative	2,6	3E-04	8E-07
EHI_159640	iron hydrogenase, putative	2,6	2E-04	4E-07
EHI_109220	proteoglycan-4 precursor, putative	2,6	5E-04	4E-06
EHI_155220	T-complex protein 1 subunit alpha, putative	2,5	9E-04	1E-05
EHI_033680	valyl-tRNA synthetase, putative [6.1.1.9]	2,5	3E-04	1E-06
EHI_147700	ubiquitin-conjugating enzyme family protein	2,5	4E-04	3E-06
EHI_140720	myosin heavy chain	2,5	2E-04	3E-07
EHI_080220	Niemann-Pick C1 protein, putative	2,5	3E-04	1E-06
EHI_153810	RNA recognition motif domain containing protein	2,5	6E-04	7E-06
EHI_065330	Gal/GalNAc lectin subunit Igl2	2,5	8E-03	3E-04
EHI_076810	serine/threonine- protein phosphatase PP-Z, putative	2,5	3E-04	7E-07
EHI_167520	60S ribosomal protein L3, putative	2,5	1E-03	3E-05
EHI_088020	alcohol dehydrogenase, putative	2,5	3E-04	2E-06
EHI_080890	26S protease regulatory subunit 7, putative	2,5	4E-04	2E-06
EHI_192460	diaphanous protein, homolog 1, putative	2,5	5E-04	4E-06
EHI_167840	WH2 motif domain contaning protein	2,5	2E-03	7E-05
EHI_146400	phospholipase D, putative	2,5	4E-04	3E-06
EHI_073630	serine-threonine-isoleucine rich protein, putative (Eh STIRP)	2,5	9E-04	2E-05
EHI_054800	SH3 domain protein	2,5	4E-04	3E-06
EHI_049570	RhoGAP domain containing protein	2,5	9E-04	1E-05
47.t00016	pyruvate phosphate dikinase	2,5	3E-04	8E-07
EHI_111060	DNA ligase, putative	2,5	3E-04	1E-06
EHI_022980	ubiquitin-like protein	2,5	4E-04	3E-06
EHI_067970	chorein, putative	2,5	2E-04	4E-07
EHI_173410	mucin-2 precursor, putative	2,5	6E-04	8E-06
EHI_187280	transcription initiation factor SPT5, putative	2,5	6E-04	7E-06
EHI_047810	lysyl-tRNA synthetase, putative	2,5	6E-04	8E-06
EHI_158020	transcription initiation factor IIIB chain BRF, putative	2,5	5E-04	5E-06

EHI_049770	mitotic chromosome and X-chromosome-associated protein, putative	2,5	6E-04	7E-06
EHI_198010	19S cap proteasome S2 subunit	2,5	1E-03	2E-05
EHI_118900	vacuolar sorting protein VPS4, putative	2,5	6E-04	6E-06
EHI_110150	zinc/cadmium transporter, putative	2,5	3E-04	6E-07
EHI_126170	cysteine protease, putative	2,5	2E-04	5E-07
EHI_175600	heat shock protein 70, mitochondrial, putative	2,4	3E-04	1E-06
EHI_125910	double-strand break repair protein MRE11, putative	2,4	8E-04	1E-05
EHI_072050	Viral A-type inclusion protein repeat, putative	2,4	3E-04	9E-07
EHI_044480	WD domain containing protein	2,4	5E-04	3E-06
EHI_081930	ATPase, AAA family protein (Pfam:AAA.hmm,sc)	2,4	3E-04	9E-07
EHI_101120	heat shock protein 70, mitochondrial, putative	2,4	3E-04	9E-07
EHI_180320	tyrosine kinase, putative	2,4	6E-04	8E-06
EHI_065320	heat shock protein 70, putative	2,4	2E-04	5E-07
EHI_092100	chitinase, putative	2,4	5E-03	2E-04
530.t00002	mitochondrial type hsp70, putative	2,4	2E-04	4E-07
EHI_174070	dTDP-D-glucose 4,6-dehydratase, putative	2,4	5E-04	4E-06
EHI_166870	GTP binding protein, putative	2,4	5E-04	4E-06
EHI_151440	cysteine proteinase, putative	2,4	5E-04	5E-06
EHI_035940	Rho GTPase activating protein, putative	2,4	5E-04	4E-06
EHI_110180	myosin heavy chain	2,4	2E-04	4E-07
EHI_066510	protein kinase, putative	2,4	3E-04	1E-06
EHI_177160	signal peptide peptidase family protein	2,4	6E-04	7E-06
EHI_196560	dual specificity protein phosphatase, putative	2,4	3E-04	1E-06
EHI_107550	bacterial transferase hexapeptide family protein	2,4	3E-04	1E-06
EHI_004840	bacterial transferase hexapeptide family protein	2,4	1E-03	2E-05
EHI_056450	Rho GTPase activating protein, putative	2,4	6E-04	8E-06
EHI_110750	dipeptidyl-peptidase III, putative	2,3	4E-04	2E-06
415.t00003	actin binding protein, putative	2,3	8E-04	1E-05
EHI_183900	snoRNA binding protein, putative	2,3	5E-04	4E-06
346.t00002	alcohol dehydrogenase 3, putative	2,3	2E-04	4E-07
EHI_167130	filopodin, putative	2,3	8E-04	1E-05
EHI_001950	heat shock protein 70 family	2,3	3E-04	1E-06
174.t00002	mitochondrial type hsp70, putative	2,3	6E-04	7E-06
EHI_037230	chaperone protein dnaK, putative	2,3	1E-03	2E-05
EHI_045090	pyruvate phosphate dikinase	2,3	5E-04	3E-06
EHI_090000	proteasome alpha subunit, putative	2,3	3E-04	1E-06
EHI_192610	sericin 1 precursor, putative	2,3	1E-03	2E-05
EHI_013760	heat shock protein 70, mitochondrial, putative	2,3	3E-04	2E-06
EHI_103840	DNA repair protein, putative (S.cerevisiae)	2,3	5E-04	6E-06
EHI_086500	short chain dehydrogenase family protein	2,3	4E-04	2E-06
EHI_132370	ccaat-box-binding transcription factor, putative	2,3	5E-04	4E-06
EHI_164370	putative GTPase	2,3	3E-03	9E-05
EHI_137710	RNA recognition motif domain containing protein	2,3	5E-04	5E-06
EHI_012600	metallo-beta-lactamase family protein	2,3	2E-04	4E-07
EHI_127700	heat shock protein 70, mitochondrial, putative	2,3	6E-04	8E-06
EHI_130700	enolase, putative (eno) [4.2.1.11]	2,3	2E-03	5E-05
EHI_006820	importin alpha, putative	2,3	2E-03	4E-05
EHI_187110	Rho GTPase activating protein, putative	2,3	7E-04	1E-05
EHI_035550	regulator of nonsense transcripts, putative	2,3	1E-03	3E-05
EHI_009570	gelsolin repeat protein, putative	2,2	2E-04	5E-07
EHI_194400	Charged multivesicular body protein, putative	2,2	1E-03	2E-05
EHI_047750	Nucleotide-binding protein , putative (NBP1)	2,2	3E-04	8E-07
EHI_080100	cylicin-2, putative	2,2	2E-03	4E-05
EHI_155520	glutamine synthetase, putative [6.3.1.2]	2,2	3E-03	1E-04
EHI_158060	RNA-binding protein, putative	2,2	3E-04	7E-07
34.t00018	developmentally regulated protein, putative	2,2	1E-03	2E-05
EHI_052840	RhoGAP domain containing protein	2,2	6E-04	7E-06
523.t00003	enolase, putative	2,2	8E-04	1E-05
EHI_149900	Rab GTPase activating protein, putative	2,2	2E-04	5E-07
EHI_038920	DNA topoisomerase III, putative	2,2	4E-04	2E-06
39.t00001	alcohol dehydrogenase 3, putative	2,2	7E-04	9E-06
EHI_157020	maltose O-acetyltransferase, putative	2,2	5E-04	4E-06
EHI_192760	copine, putative	2,2	7E-04	9E-06
EHI_196550	Rap/Ran GTPase activating protein	2,2	3E-04	1E-06
70.t00021	S-adenosylmethionine synthetase, putative	2,2	2E-03	6E-05
72.t00042	alcohol dehydrogenase 3, putative	2,2	4E-04	2E-06
EHI_016010	66 kDa stress protein, putative	2,2	5E-04	5E-06
EHI_018740	peroxiredoxin family protein	2,2	6E-04	8E-06
209.t00010	glucose-6-phosphate isomerase, putative	2,2	3E-04	1E-06
EHI_007150	mitochondrial-type heat shock protein 70	2,2	3E-04	1E-06
EHI_158610	Activator 1 140 kDa subunit, putative	2,2	5E-04	4E-06
EHI_040310	Rab family GTPase	2,2	8E-04	1E-05

EHI_140670	DNA replication licensing factor, putative (proteinforMGC:1)	2,2	5E-04	5E-06
EHI_017760	tyrosine kinase, putative	2,2	9E-04	2E-05
EHI_118110	Acid sphingomyelinase-like phosphodiesterase, putative	2,2	7E-03	3E-04
EHI_030180	alcohol dehydrogenase 3, putative	2,2	5E-04	5E-06
EHI_011920	protein kinase, putative	2,2	1E-03	2E-05
18.t00006	WD repeat protein	2,2	5E-04	5E-06
18.t00004	WD repeat protein	2,2	2E-03	4E-05
EHI_060350	splicing factor Prp8, putative	2,2	9E-04	2E-05
EHI_125700	dTDP-D-glucose 4,6-dehydratase, putative	2,2	3E-04	6E-07
EHI_152180	RNA recognition motif domain containing protein	2,2	3E-04	2E-06
EHI_178960	acetyl-CoA synthetase, putative	2,2	5E-04	5E-06
EHI_190460	amino acid transporter, putative	2,1	7E-03	3E-04
EHI_148910	interaptin, putative	2,1	3E-04	8E-07
EHI_183110	RNA recognition motif domain containing protein	2,1	3E-03	1E-04
EHI_004340	serine-threonine-isoleucine rich protein, putative (Eh STIRP)	2,1	3E-04	1E-06
EHI_180380	heat shock protein 70, putative	2,1	1E-03	3E-05
96.t00030	protein phosphatase 2C, putative	2,1	5E-04	3E-06
EHI_000780	chromodomain-helicase-DNA-binding protein, putative	2,1	3E-04	1E-06
70.t00028	S-adenosylmethionine synthetase, putative	2,1	3E-03	1E-04
EHI_135100	zinc finger domain containing protein	2,1	5E-04	4E-06
262.t00004	aspartyl-tRNA synthetase, putative	2,1	2E-03	5E-05
EHI_140330	protein kinase, putative	2,1	1E-03	2E-05
326.t00003	alcohol dehydrogenase 3, putative	2,1	6E-04	6E-06
EHI_011390	Rho guanine nucleotide exchange factor, putative	2,1	1E-03	3E-05
EHI_007480	villin, putative	2,1	6E-04	7E-06
EHI_045600	Ras family protein	2,1	3E-04	1E-06
38.t00004	reverse transcriptase, putative	2,1	3E-03	1E-04
70.t00027	S-adenosylmethionine synthetase, putative	2,1	2E-03	6E-05
EHI_150430	villin-related protein	2,1	3E-04	1E-06
3.t00096	malic enzyme	2,1	4E-04	2E-06
EHI_180450	structural maintenance of chromosomes protein	2,1	4E-04	2E-06
EHI_049640	phospholipid-transporting P-type ATPase, putative	2,1	3E-04	1E-06
EHI_080280	glucosamine 6-phosphate N-acetyltransferase. putative [2.3.1.4]	2,1	1E-03	2E-05
72.t00005	protein kinase, putative	2,1	9E-04	2E-05
451.t00004	AIG1 family protein, putative	2,1	6E-04	6E-06
41.t00044	DNA-directed RNA polymerase I largest subunit, putative	2,1	3E-04	7E-07
EHI_001400	RAD23 protein, putative	2,1	5E-04	4E-06
EHI_153830	midasin, putative	2,1	3E-04	8E-07
EHI_078260	HEAT repeat domain containing protein	2,1	3E-04	1E-06
28.t00064	DnaJ family protein	2,1	1E-03	2E-05
EHI_103900	nucleosome-binding protein 1, putative	2,1	1E-03	3E-05
EHI_055960	ubiquitin fusion degradation protein, putative	2,1	3E-04	1E-06
EHI_047730	glucose-6-phosphate isomerase, putative	2,1	3E-04	9E-07
EHI_078170	protein kinase, putative	2,1	3E-04	1E-06
EHI_048860	U3 small nucleolar ribonucleo protein protein MPP10, putative	2,1	4E-04	2E-06
146.t00027	predicted protein	2,1	8E-04	1E-05
EHI_156390	casein kinase 1, putative	2,1	4E-04	2E-06
EHI_119250	tyrosine kinase, putative	2,1	7E-04	1E-05
376.t00007	S-adenosylmethionine synthetase, putative	2,1	4E-03	1E-04
31.t00044	Arf GTPase activating protein, putative	2,1	3E-03	9E-05
EHI_059670	Rab family GTPase	2,1	5E-04	4E-06
EHI_078250	vacuolar sorting protein 26, putative	2,1	1E-03	2E-05
EHI_117970	DNA replication licensing factor	2,1	4E-04	2E-06
EHI_195110	S-adenosylmethionine synthetase, putative	2,1	5E-03	2E-04
69.t00007	60S ribosomal protein L3, putative	2,0	5E-04	4E-06
EHI_093860	MIT domain protein	2,0	1E-03	2E-05
EHI_155170	DNA mismatch repair protein PMS1, putative	2,0	3E-04	1E-06
EHI_141560	Ras family protein	2,0	3E-04	8E-07
EHI_088430	DNA repair helicase, putative	2,0	3E-04	1E-06
313.t00002	PH domain protein	2,0	1E-03	3E-05
7.t00076	OFR 1.5 protein-related	2,0	7E-04	1E-05
EHI_160750	cysteine surface protein, putative	2,0	4E-03	1E-04
117.t00026	60S ribosomal protein L3, putative	2,0	5E-04	5E-06
EHI_072240	glucose-6-phosphate isomerase, putative	2,0	5E-04	4E-06
EHI_164750	26S proteasome non-ATPase regulatory subunit 14, putative	2,0	8E-04	1E-05
EHI_135070	RNA recognition motif domain containing protein	2,0	5E-04	5E-06
470.t00004	translation elongation factor EF-1 alpha, putative	2,0	1E-03	2E-05
EHI_153670	U1 small nuclear ribonucleoprotein subunit, putative	2,0	4E-04	3E-06
EHI_138300	bromodomain protein, putative	2,0	6E-04	6E-06
EHI_000900	ThiF family protein	2,0	3E-04	1E-06
EHI_054790	tRNA nucleotidyltransferase, putative	2,0	1E-03	3E-05
EHI_045440	ubiquitin carboxyl-terminal hydrolase, putative	2,0	4E-04	2E-06

EHI_197540	protein kinase, putative	2,0	3E-04	1E-06
EHI_110980	Rho guanine nucleotide exchange factor, putative	2,0	3E-04	2E-06
EHI_124430	Zn-dependent peptidase, putative	2,0	2E-03	5E-05
3.t00052	glutamate synthase small subunit, putative	2,0	3E-04	1E-06
EHI_183280	DnaJ family protein	2,0	6E-04	7E-06
EHI_200820	ubiquitin carboxyl-terminal hydrolase, putative	2,0	2E-03	4E-05
EHI_049620	Fe-S cluster assembly protein NifU, putative	2,0	8E-03	4E-04
