# Supplementary material for: Endoplasmic Reticulum Stress-Sensing Mechanism Is Activated in Entamoeba histolytica upon Treatment with Nitric Oxide
Source: PLoS One. 2012 Feb 24;7(2):e31777. doi: 10.1371/journal.pone.0031777 (PMC3286455; doi:10.1371/journal.pone.0031777)
Supplement: Table S2 — Genes downregulated by NO treatment. (PDF) [file pone.0031777.s005.pdf]

Table S2. Genes downregulated by NO treatment

GENE ID	Description	FC	BY	rawp
EHI_172850	surface antigen ariel1, putative	0,23	4E-04	2E-06
EHI_169800	surface antigen ariel1, putative	0,23	5E-04	4E-06
EHI_186470	surface antigen ariel1, putative	0,26	4E-04	3E-06
EHI_156310	ribonuclease, putative	0,27	4E-04	3E-06
EHI_080200	surface antigen ariel1, putative	0,27	3E-04	2E-06
EHI_062790	thioredoxin, putative	0,28	6E-04	7E-06
EHI_165270	L-myo-inositol-1-phosphate synthase, putative	0,28	2E-04	9E-08
EHI_070720	L-myo-inositol-1-phosphate synthase	0,29	2E-04	5E-07
273.t00008	L-myo-inositol-1-phosphate synthase	0,31	3E-04	6E-07
EHI_131360	surface antigen ariel1, putative	0,32	2E-04	3E-07
EHI_095820	ATP-binding cassette, putative	0,33	5E-04	4E-06
EHI_068110	Ras guanine nucleotide exchange factor, putative	0,33	2E-04	4E-07
EHI_169300	ribonuclease, putative	0,33	2E-04	2E-07
EHI_067590	troponin-like protein, putative	0,34	3E-04	7E-07
EHI_193350	3'(2'),5'-bisphosphate nucleotidase, putative [3.1.3.7]	0,35	2E-04	5E-07
108.t00027	L-myo-inositol-1-phosphate synthase	0,36	2E-04	4E-07
611.t00003	surface antigen ariel1-related	0,36	3E-04	7E-07
EHI_030600	zinc transporter, putative	0,37	3E-04	2E-06
EHI_132430	PQ loop repeat protein	0,37	4E-04	2E-06
EHI_141840	calmodulin, putative	0,38	5E-04	4E-06
EHI_062840	UDP-N-acetylglucosamine--dolichyl-phosphate N-acetylglucosaminephosphotransferase, putative	0,38	3E-04	9E-07
EHI_153380	membrane transporter, putative	0,39	2E-04	6E-07
EHI_124580	UDP-N-acetylglucosamine transporter, putative	0,39	2E-04	5E-07
EHI_099240	clathrin-adaptor medium chain, putative	0,39	5E-04	5E-06
EHI_194500	ADP-ribosylation factor, putative	0,40	2E-04	2E-07
EHI_104360	nucleoside diphosphate kinase, putative	0,40	4E-04	3E-06
EHI_044610	ribosome biogenesis protein NEP1, putative	0,40	3E-04	9E-07
EHI_065870	RNA-binding protein, putative (proteinforIMAGE)	0,40	4E-04	2E-06
EHI_079300	long-chain-fatty-acid--CoA ligase, putative	0,41	3E-04	7E-07
EHI_045480	high mobility group (HMG) box domain containing protein	0,41	6E-04	6E-06
EHI_105060	geranylgeranyl pyrophosphate synthase, putative	0,41	6E-04	7E-06
EHI_093870	phosphoglycerate mutase family protein	0,42	3E-04	1E-06
EHI_167080	acetyltransferase, GNAT family	0,42	3E-04	1E-06
EHI_005060	Fe-hydrogenase, putative	0,42	3E-03	8E-05
EHI_060330	peptidase S54 (rhomboid) family protein	0,42	5E-04	4E-06
EHI_142150	BTN1 protein, putative	0,42	3E-04	1E-06
EHI_118270	amoebapore C	0,43	3E-04	8E-07
EHI_186480	translation initiation factor eIF-5A, putative (eif5A)	0,43	3E-04	1E-06
EHI_121860	microsomal signal peptidase subunit, putative	0,43	2E-04	4E-07
EHI_148480	WD domain containing protein	0,43	2E-04	3E-07
EHI_127160	ribonuclease, putative	0,43	5E-04	5E-06
139.t00015	3'(2'),5'-bisphosphate nucleotidase, putative	0,43	5E-04	3E-06
EHI_166410	myb-like DNA-binding domain containing protein	0,44	7E-04	1E-05
EHI_068750	vacuolar ATP synthase subunit H, putative	0,44	2E-04	4E-07
EHI_093900	eukaryotic initiation factor 4A, putative	0,44	2E-04	5E-07
EHI_148470	aspartate--ammonia ligase, putative (asnA) [6.3.1.1]	0,44	5E-04	5E-06
EHI_103550	Clathrin adaptor complex small chain, putative	0,44	3E-04	7E-07
EHI_141990	60S ribosomal protein L7, putative	0,44	1E-03	2E-05
EHI_147570	Rho GDP exchange inhibitor, putative	0,44	3E-04	6E-07
41.t00016	ABC transporter, putative	0,45	1E-03	2E-05
EHI_125160	guanine nucleotide exchange factor, putative	0,45	5E-04	6E-06
EHI_050140	casein kinase, putative	0,45	3E-04	7E-07
EHI_067920	CDP-alcohol phosphatidyltransferase family protein	0,45	1E-03	2E-05
EHI_175490	dual specificity protein phosphatase, putative	0,45	3E-04	1E-06
EHI_175020	peptidase, putative	0,45	5E-04	3E-06
86.t00002	peptidase, putative	0,45	7E-04	9E-06
EHI_133970	thioredoxin, putative	0,45	4E-04	2E-06
217.t00002	geranylgeranyl pyrophosphate synthase, putative	0,45	9E-04	2E-05
EHI_151290	longevity-assurance family protein	0,46	4E-04	2E-06
EHI_017720	zinc finger protein, putative	0,46	3E-04	1E-06
EHI_007920	ankyrin repeat protein, putative	0,46	9E-04	1E-05
EHI_103450	actin binding protein, putative	0,46	3E-04	9E-07
260.t00003	elongation factor 1 beta, putative	0,46	2E-04	5E-07
325.t00008	geranylgeranyl pyrophosphate synthase, putative	0,46	6E-04	7E-06

EHI_102280	H/ACA ribonucleoprotein complex subunit 2-like protein, putative	0,46	1E-03	2E-05
EHI_193360	histone H3, putative	0,47	3E-04	8E-07
EHI_126010	ribosome biogenesis protein, putative	0,47	4E-04	3E-06
EHI_132650	cysteine proteinase 2 precursor, putative	0,47	7E-04	1E-05
EHI_102940	60S acidic ribosomal protein PO, putative	0,47	1E-03	2E-05
EHI_049430	protein tyrosine phosphatase, putative	0,47	4E-04	3E-06
EHI_131470	ribosome biogenesis protein Nop10, putative	0,47	1E-03	2E-05
EHI_135740	acyl-CoA synthetase, putative	0,47	3E-04	6E-07
EHI_192590	beta-amylase, putative	0,47	7E-04	1E-05
EHI_134640	acetyltransferase, putative	0,47	6E-04	6E-06
EHI_127260	PH domain protein	0,47	8E-04	1E-05
EHI_148410	PQ loop repeat protein	0,47	1E-03	2E-05
EHI_010850	cysteine proteinase, putative	0,48	4E-04	3E-06
253.t00001	phosphoserine aminotransferase, putative	0,48	3E-04	2E-06
EHI_083570	nuclear complex protein 4, putative	0,48	4E-04	3E-06
EHI_142720	geranylgeranyl pyrophosphate synthase, putative	0,48	9E-04	2E-05
EHI_189960	ADP-ribosylation factor, putative	0,48	1E-03	3E-05
EHI_140640	F-actin capping protein subunit alpha, putative	0,48	3E-04	1E-06
EHI_092190	fatty acid elongase, putative	0,48	7E-04	8E-06
EHI_159790	60S acidic ribosomal protein PO, putative	0,48	7E-04	1E-05
EHI_169660	lipid phosphatase, putative	0,48	6E-04	6E-06
EHI_124530	HEAT repeat domain containing protein	0,48	5E-04	4E-06
EHI_177580	sodium/sulfate symporter, putative	0,49	4E-04	2E-06
EHI_073580	leucine-rich repeat containing protein	0,49	5E-03	2E-04
EHI_177520	Rab family GTPase	0,49	3E-04	2E-06
EHI_096400	F-box domain containing membrane protein, putative	0,49	5E-04	6E-06
EHI_163510	exosome complex exonuclease RRP4, putative	0,49	3E-04	2E-06
EHI_169350	nonpathogenic pore-forming peptide precursor, putative	0,49	6E-03	3E-04
EHI_125740	oxidoreductase, putative	0,49	6E-04	8E-06
EHI_035750	lecithin:cholesterol acyltransferase domain-containing protein	0,49	3E-04	1E-06
EHI_122780	DNA-directed RNA polymerase subunit N, putative	0,50	1E-03	2E-05
EHI_148890	calmodulin, putative	0,50	6E-04	8E-06
EHI_137900	DNA-directed RNA polymerase I subunit 13, putative	0,50	6E-04	7E-06
EHI_039610	cysteine proteinase, putative	0,50	5E-04	5E-06
EHI_069970	calcineurin catalytic subunit A, putative	0,50	4E-04	3E-06
EHI_169580	nucleoside transporter, putative	0,50	5E-04	5E-06
EHI_109680	START domain protein	0,50	5E-04	4E-06
EHI_148520	Mob1/phocein family protein	0,50	4E-04	2E-06
EHI_156430	NUF1 protein, putative	0,50	9E-04	2E-05
