# Supplementary material for: Endoplasmic Reticulum Stress-Sensing Mechanism Is Activated in Entamoeba histolytica upon Treatment with Nitric Oxide
Source: PLoS One. 2012 Feb 24;7(2):e31777. doi: 10.1371/journal.pone.0031777 (PMC3286455; doi:10.1371/journal.pone.0031777)
Supplement: Table S3 — GOSlim Terms of Genes modulated by NO treatment. The data were analyzed using GO Slim Terms implemented in AmiGO (http://amigo.geneontology.org/cgi-bin/amigo/slimmer), based on the GO annotations in AmoebaDB version 1.3. (http://amoebadb.org/amoeba/). (PDF) [file pone.0031777.s006.pdf]

Suplemmental Table3. GOSlim Terms of Genes modulated by NO treatment

GOSlim Terms	Number of Genes	Fraction of Whole Genome GOSlim Terms
**UP REGULATED GENES**		
GO:0005488 binding	110	0,0466
GO:0003824 catalytic activity	108	0,0535
GO:0016787 hydrolase activity	48	0,0543
GO:0000166 nucleotide binding	39	0,0389
GO:0016491 oxidoreductase activity	29	0,1457
GO:0005515 protein binding	21	0,0355
GO:0043167 ion binding	17	0,0430
GO:0016740 transferase activity	16	0,0233
GO:0003676 nucleic acid binding	15	0,0272
GO:0008233 peptidase activity	6	0,0531
GO:0048037 cofactor binding	6	0,0968
GO:0044464 cell part	6	0,0035
GO:0016874 ligase activity	4	0,0417
GO:0030234 enzyme regulator activity	4	0,0123
GO:0051540 metal cluster binding	4	0,2000
GO:0005622 intracellular	4	0,0028
GO:0005215 transporter activity	3	0,0196
GO:0016829 lyase activity	3	0,0732
GO:0016853 isomerase activity	3	0,0484
GO:0019842 vitamin binding	3	0,1429
GO:0004386 helicase activity	2	0,0333
GO:0008641 small protein activating enzyme activity	2	0,5000
GO:0016020 membrane	2	0,0051
GO:0008152 metabolic process	2	0,0010
GO:0009987 cellular process	2	0,0009
GO:0044237 cellular metabolic process	2	0,0013
GO:0004871 signal transducer activity	1	0,0769
GO:0005198 structural molecule activity	1	0,0043
GO:0008144 drug binding	1	0,2500
GO:0008289 lipid binding	1	0,0500
GO:0030528 transcription regulator activity	1	0,0667
GO:0060089 molecular transducer activity	1	0,0769
GO:0005634 nucleus	1	0,0041
GO:0005681 spliceosomal complex	1	0,2500
GO:0030529 ribonucleoprotein complex	1	0,0044
GO:0032991 macromolecular complex	1	0,0023
GO:0043226 organelle	1	0,0017
GO:0043229 intracellular organelle	1	0,0017
GO:0043231 intracellular membrane-bounded organelle	1	0,0032
GO:0006464 protein modification process	1	0,0021
GO:0019538 protein metabolic process	1	0,0010
GO:0043170 macromolecule metabolic process	1	0,0008
GO:0043412 macromolecule modification	1	0,0020
GO:0044238 primary metabolic process	1	0,0006
GO:0051186 cofactor metabolic process	1	0,0417

DOWN REGULATED GENES		
GO:0003824 catalytic activity	31	0,0154
GO:0005488 binding	25	0,0106
GO:0016787 hydrolase activity	16	0,0181
GO:0016740 transferase activity	10	0,0146
GO:0005215 transporter activity	7	0,0458
GO:0044464 cell part	5	0,0029
GO:0000166 nucleotide binding	5	0,0050
GO:0003676 nucleic acid binding	5	0,0091
GO:0005515 protein binding	5	0,0085
GO:0008233 peptidase activity	5	0,0442
GO:0016020 membrane	3	0,0076
GO:0030234 enzyme regulator activity	3	0,0092
GO:0043167 ion binding	3	0,0076
GO:0005622 intracellular	2	0,0014
GO:0043226 organelle	2	0,0034
GO:0043229 intracellular organelle	2	0,0034
GO:0043231 intracellular membrane-bounded organelle	2	0,0064
GO:0009987 cellular process	1	0,0004
GO:0065007 biological regulation	1	0,0014
GO:0005634 nucleus	1	0,0041
GO:0005783 endoplasmic reticulum	1	0,0400
GO:0004386 helicase activity	1	0,0167
GO:0005198 structural molecule activity	1	0,0043
GO:0008289 lipid binding	1	0,0500
GO:0016491 oxidoreductase activity	1	0,0050
GO:0016853 isomerase activity	1	0,0161
GO:0016874 ligase activity	1	0,0104
GO:0030528 transcription regulator activity	1	0,0667
GO:0043021 ribonucleoprotein binding	1	0,2000
GO:0048037 cofactor binding	1	0,0161
GO:0051540 metal cluster binding	1	0,0500
