# Supplementary material for: Endoplasmic Reticulum Stress-Sensing Mechanism Is Activated in Entamoeba histolytica upon Treatment with Nitric Oxide
Source: PLoS One. 2012 Feb 24;7(2):e31777. doi: 10.1371/journal.pone.0031777 (PMC3286455; doi:10.1371/journal.pone.0031777)
Supplement: Table S4 — Genes modulated by treatment with nitric oxide. (PDF) [file pone.0031777.s007.pdf]

Table S4. GENES MODULATED BY TREATMENT WITH NITRIC OXIDE				
A. GENES ENCODING HEAT SHOCK PROTEINS				
GENE ID	Description	FC	BY	RAWP
**HSP70**				
EHI_061640	heat shock protein70, hsp70A2	33,7	6,6E-04	8,5E-06
EHI_132540	heat shock protein70, hsp70A2	29,3	5,3E-04	5,2E-06
EHI_188610	heat shock protein 70	23,4	4,7E-04	3,9E-06
EHI_192440	heat shock protein70, hsp70A2	22,1	8,3E-04	1,3E-05
EHI_132530	heat shock protein70, hsp70A2	21,8	6,1E-04	7,2E-06
EHI_015390	heat shock protein70, hsp70A2	16,6	4,2E-04	2,8E-06
EHI_108130	heat shock protein70, hsp70A2	16	5,6E-04	5,9E-06
EHI_026590	heat shock protein 70	14,9	5,8E-04	6,2E-06
EHI_104330	heat shock protein 70	11,7	6,2E-04	7,6E-06
EHI_197860	heat shock protein 70	10	2,2E-04	2,8E-07
EHI_052860	heat shock protein 70	6,7	1,5E-04	3,6E-08
EHI_155490	heat shock protein 70	3,1	3,8E-04	2,2E-06
EHI_166050	mitochondrial type hsp70	3	2,7E-04	8,2E-07
EHI_100810	mitochondrial type hsp 70	2,9	2,7E-04	8,7E-07
EHI_175600	mitochondrial type hsp 70	2,4	2,9E-04	1,0E-06
EHI_101120	mitochondrial type hsp 70	2,4	2,8E-04	9,4E-07
EHI_065320	heat shock protein 70	2,4	2,4E-04	5,1E-07
EHI_001950	heat shock protein 70	2,3	2,9E-04	1,1E-06
EHI_013760	mitochondrial type hsp 70	2,3	3,3E-04	1,5E-06
EHI_127700	mitochondrial type hsp 70	2,3	6,4E-04	8,0E-06
EHI_007150	mitochondrial type hsp 70	2,2	2,9E-04	1,1E-06
EHI_180380	heat shock protein 70	2,1	1,4E-03	3,2E-05
**HSP101/ClpB**				
EHI_072140	heat shock protein 101	66,6	1,7E-04	6,8E-08
EHI_183680	AAA family ATPase	55,8	1,7E-04	8,1E-08
64.t00034	heat shock protein 101	43,5	2,7E-04	7,1E-07
EHI_155060	chaperone clpB	37,3	1,9E-04	1,2E-07
EHI_090840	chaperone clpB	36,4	2,4E-04	5,1E-07
EHI_076480	heat shock protein 101	36,4	1,9E-04	1,0E-07
EHI_094680	chaperone clpB	36,3	2,3E-04	3,3E-07
81.t00001	heat shock protein 101	32,4	1,8E-04	9,5E-08
EHI_063440	heat shock protein 101	30	2,2E-04	2,4E-07
EHI_094470	heat shock protein 101	27,2	2,2E-04	2,3E-07
EHI_013550	heat shock protein 101	26,5	2,7E-04	7,1E-07
EHI_178230	heat shock protein 101	25,3	2,2E-04	2,3E-07
EHI_090840	chaperone clpB	24,7	5,8E-04	6,2E-06
**HSP90**				
EHI_102270	heat shock protein 90	9,9	4,2E-05	1,1E-09
EHI_196940	heat shock protein 90	7,9	5,5E-05	4,0E-09
**OTHERS**				
EHI_133220	protein with DnaJ and myb domains	4,1	4,5E-04	3,4E-06
EHI_128200	protein with DnaJ and myb domains	3,6	1,9E-04	1,2E-07
EHI_178850	peptidyl-prolyl cis-trans isomerase	5	2,3E-04	4,2E-07
EHI_158050	Hsc70-interacting protein Hip	3,9	2,3E-04	3,2E-07
EHI_055680	heat shock protein, Hsp20 family	15,9	4,6E-04	3,8E-06
EHI_193390	heat shock protein, Hsp20 family	3,9	1,9E-04	1,5E-07
EHI_125830	heat shock protein, Hsp20 family	2,9	5,7E-04	6,1E-06
EHI_187000	DnaJ family protein	9,6	5,0E-04	4,7E-06
EHI_151260	DnaJ family protein	6,3	1,7E-04	7,2E-08
EHI_183280	DnaJ family protein	2,1	9,6E-04	1,7E-05
EHI_125800	chaperonin-containing TCP-1 zeta	2,8	6,8E-04	9,1E-06
B.. GENES LINKED TO PROTEASOME ACTIVITIES				
GENE ID	Description	FC	BY	RAWP
EHI_020270	ubiquitin-activating enzyme	6,6	1,7E-04	8,2E-08
EHI_049680	26S proteasome regulatory subunit	3,8	1,9E-04	1,1E-07
EHI_187180	ubiquitin-ligase	3,3	4,3E-04	3,0E-06
EHI_137730	ubiquitin carboxyl-terminal hydrolase	3,1	1,8E-03	4,6E-05
EHI_177320	26S proteasome subunit P45	2,9	3,0E-04	1,2E-06
251.t00015	26S proteasome subunit P45	2,8	2,9E-04	1,0E-06

EHI_038690	ubiquitin-activating enzyme E1 1	2,7	8,2E-04	1,3E-05
EHI_098550	ubiquitin-activating enzyme	2,6	2,2E-04	2,2E-07
EHI_147700	ubiquitin-conjugating enzyme	2,5	4,2E-04	2,8E-06
EHI_022980	ubiquitin-like protein	2,5	4,0E-04	2,5E-06
EHI_198010	19S cap proteasome S2 subunit	2,5	9,5E-04	1,7E-05
EHI_055960	ubiquitin fusion degradation protein	2,1	3,1E-04	1,3E-06
EHI_164750	26S proteasome regulatory subunit 14	2	8,1E-04	1,2E-05
EHI_045440	ubiquitin carboxyl-terminal hydrolase	2	3,5E-04	1,8E-06
EHI_200820	ubiquitin carboxyl-terminal hydrolase	2	1,7E-03	4,3E-05

C. GENES ENCODING PROTEINS INVOLVED IN DNA REPAIR

GENE ID	Description	FC	BY	rawp
EHI_023090	recQ family DNA helicase	3,9	2,1E-04	1,9E-07
EHI_076880	DNA replication licensing factor	3,3	2,9E-04	1,1E-06
EHI_094260	Exonuclease I	3,3	1,7E-04	7,0E-08
EHI_054240	DNA repair helicase	3,2	2,8E-04	8,9E-07
EHI_031220	DNA repair protein RAD51	2,9	3,8E-04	2,2E-06
EHI_120640	DNA topoisomerase II	2,8	2,7E-04	7,1E-07
EHI_076880	DNA replication licensing factor	2,7	2,3E-04	4,2E-07
EHI_050760	mutT/nudix family protein	2,6	2,0E-04	1,6E-07
EHI_111060	DNA ligase	2,5	3,1E-04	1,3E-06
EHI_125910	double-strand break repair protein MRE11	2,4	8,2E-04	1,3E-05
EHI_103840	DNA repair protein	2,3	5,5E-04	5,6E-06
EHI_038920	DNA topoisomerase III	2,2	3,5E-04	1,8E-06
EHI_140670	DNA replication licensing factor, putative	2,2	5,0E-04	4,6E-06
EHI_001400	RAD23 protein, putative	2,1	4,7E-04	3,9E-06
EHI_117970	DNA replication licensing factor	2,1	3,7E-04	2,1E-06
EHI_155170	DNA mismatch repair protein PMS1	2,0	2,9E-04	1,0E-06
EHI_088430	DNA repair helicase	2,0	3,2E-04	1,5E-06
EHI_129310	DNA repair/transcription protein Mms19	2,0	2,9E-04	1,1E-06
EHI_187720	DNA replication licensing factor	1,9	7,8E-04	1,2E-05
294.t00002	DNA repair and recombination protein RAD26	1,8	4,3E-04	2,9E-06
EHI_129950	DNA mismatch repair protein MLH1	1,7	9,3E-03	4,3E-04
EHI_037680	DNA polymerase, putative	1,7	2,3E-03	6,8E-05
EHI_099850	replication factor C familiy protein	1,7	6,6E-03	2,8E-04
EHI_001430	helicase domain-containing protein	1,6	7,3E-04	1,0E-05
EHI_118870	DNA replication licensing factor	1,6	1,9E-03	5,0E-05
EHI_196700	DNA polymerase, putative	1,6	1,4E-03	3,2E-05
EHI_012470	helicase domain-containing protein	1,6	2,3E-03	6,5E-05
EHI_151520	DNA polymerase alpha catalytic subunit	1,6	2,5E-03	7,7E-05
EHI_112840	Rad52/22 family double-strand break repair	1,5	1,4E-03	3,2E-05
EHI_132860	DNA polymerase e	1,5	2,3E-03	6,5E-05
EHI_119290	recQ family helicase	1,5	5,4E-03	2,2E-04
EHI_164190	DNA polymerase	1,5	1,5E-02	7,6E-04
EHI_141120	helicase	1,4	1,6E-03	3,9E-05
EHI_193340	mutS family protein	1,4	3,2E-03	1,1E-04
EHI_062980	replication factor A protein 1	1,4	2,2E-03	6,3E-05
EHI_044890	helicase	1,4	3,2E-03	1,1E-04
EHI_103660	helicase	1,4	3,7E-03	1,3E-04
EHI_103660	helicase	1,4	3,7E-03	1,3E-04
EHI_160720	exosome complex exonuclease	1,3	9,9E-03	4,7E-04
EHI_104930	endonuclease V	1,3	8,3E-03	3,8E-04
EHI_194510	DNA topoisomerase	1,3	4,1E-02	2,5E-03
EHI_006690	DNA polymerase delta catalytic subunit	1,3	1,2E-02	5,7E-04
EHI_101390	Endonuclease V	1,3	9,5E-03	4,4E-04
40.t00017	DNA repair helicase	1,3	9,5E-03	4,4E-04
EHI_018010	DNA polymerase	1,3	3,1E-02	1,8E-03
EHI_068010	DNA polymerase zeta catalytic subunit	1,3	1,8E-02	9,4E-04
EHI_158110	DNA replication licensing factor	1,2	4,3E-02	2,7E-03
EHI_053600	helicase	1,2	2,5E-02	1,4E-03
EHI_090040	helicase	1,2	2,4E-02	1,3E-03
EHI_031270	damaged DNA binding protein	1,2	2,1E-02	1,1E-03
